# Deep sequence analysis of HIV adaptation following vertical transmission reveals the impact of immune pressure on the evolution of HIV

**DOI:** 10.1371/journal.ppat.1008177

**Published:** 2019-12-10

**Authors:** Jennifer Currenti, Abha Chopra, Mina John, Shay Leary, Elizabeth McKinnon, Eric Alves, Mark Pilkinton, Rita Smith, Louise Barnett, Wyatt J. McDonnell, Michaela Lucas, Francine Noel, Simon Mallal, Joseph A. Conrad, Spyros A. Kalams, Silvana Gaudieri

**Affiliations:** 1 School of Human Sciences, University of Western Australia, Crawley, Western Australia, Australia; 2 Institute for Immunology and Infectious Diseases, Murdoch University, Murdoch, Western Australia, Australia; 3 Department of Clinical Immunology, Royal Perth Hospital, Perth, Western Australia, Australia; 4 Division of Infectious Diseases, Department of Medicine, Vanderbilt University Medical Center, Nashville, Tennessee, United States of America; 5 School of Medicine, University of Western Australia, Crawley, Western Australia, Australia; 6 GHESKIO Centre, Port-au-prince, Haiti; 7 Department of Chemistry, Vanderbilt University, Nashville, Tennessee, United States of America; University of North Carolina at Chapel Hill, UNITED STATES

## Abstract

Human immunodeficiency virus (HIV) can adapt to an individual’s T cell immune response via genomic mutations that affect antigen recognition and impact disease outcome. These viral adaptations are specific to the host’s human leucocyte antigen (HLA) alleles, as these molecules determine which peptides are presented to T cells. As HLA molecules are highly polymorphic at the population level, horizontal transmission events are most commonly between HLA-mismatched donor/recipient pairs, representing new immune selection environments for the transmitted virus. In this study, we utilised a deep sequencing approach to determine the HIV quasispecies in 26 mother-to-child transmission pairs where the potential for founder viruses to be pre-adapted is high due to the pairs being haplo-identical at HLA loci. This scenario allowed the assessment of specific HIV adaptations following transmission in either a non-selective immune environment, due to recipient HLA mismatched to original selecting HLA, or a selective immune environment, mediated by matched donor/recipient HLA. We show that the pattern of reversion or fixation of HIV adaptations following transmission provides insight into the replicative cost, and likely compensatory networks, associated with specific adaptations *in vivo*. Furthermore, although transmitted viruses were commonly heavily pre-adapted to the child’s HLA genotype, we found evidence of *de novo* post-transmission adaptation, representing new epitopes targeted by the child’s T cell response. High-resolution analysis of HIV adaptation is relevant when considering vaccine and cure strategies for individuals exposed to adapted viruses via transmission or reactivated from reservoirs.

## Author summary

Highly mutable pathogens utilise genetic variations within T cell epitopes as a mechanism of immune escape (viral adaptation). The diversity of the human leucocyte antigen (HLA) molecules that present viral targets to T cells in human populations partially protects against rapid population-level accumulation of human immunodeficiency virus (HIV) adaptations through horizontal transmissions. In contrast, vertical transmissions occur between haplo-identical mother/child pairs, and potentially include adaptive changes through father-mother-child transmission, representing a pathway to complete pre-adaptation to HLA alleles in child hosts over only two transmission events. We utilised next-generation sequencing to examine HIV evolution in the unique setting of vertical HIV transmission. We predict the *in vivo* replicative cost and immune benefit of specific HIV adaptations that could be used to inform vaccine design and cure strategies to combat viral immune adaptation.

## Introduction

The breadth and strength of the host’s T cell immune response is one of the critical determinants of disease outcome following infection with human immunodeficiency virus (HIV; reviewed in [1]). T cell immune pressure on the virus can lead to the selection of mutations (adaptations) in the viral genome that allow the virus to subvert these T cell responses [2]. These adaptations occur within, and flanking, T cell epitopes presented by the host’s human leucocyte antigens (HLA), which disrupts the T cell receptor-epitope-HLA complex [3] often resulting in the loss of ability to recognise and respond to HIV.

Viral adaptations can occur from acute HIV infection onwards, progressing over the course of untreated infection, and are dependent on the strength of immune pressure balanced against the replicative cost effects of adaptations and any linked compensatory mutations. At the population level, these adaptations can be identified across the HIV genome as specific HLA allele-associated polymorphisms [4]. Greater HLA-associated polymorphism in an individual’s autologous virus has been associated with higher viral load and lower CD4^+^ T cell count [3, 5, 6].

Sexual transmission of HIV imposes a selection bias towards transmission of amino acids which confer the highest intrinsic viral fitness, and against fitness-impairing adaptations [7]. As a result of this selection bias, the rate of population-wide evolution of HIV progresses at a relatively slow rate [8]. However, if adaptations are transmitted they may enhance disease progression in HLA identical recipients [7, 9, 10]. Furthermore, infection with a pre-adapted virus is likely to compromise the initial and long-term anti-HIV T cell response in an individual.

**Fig 1:**
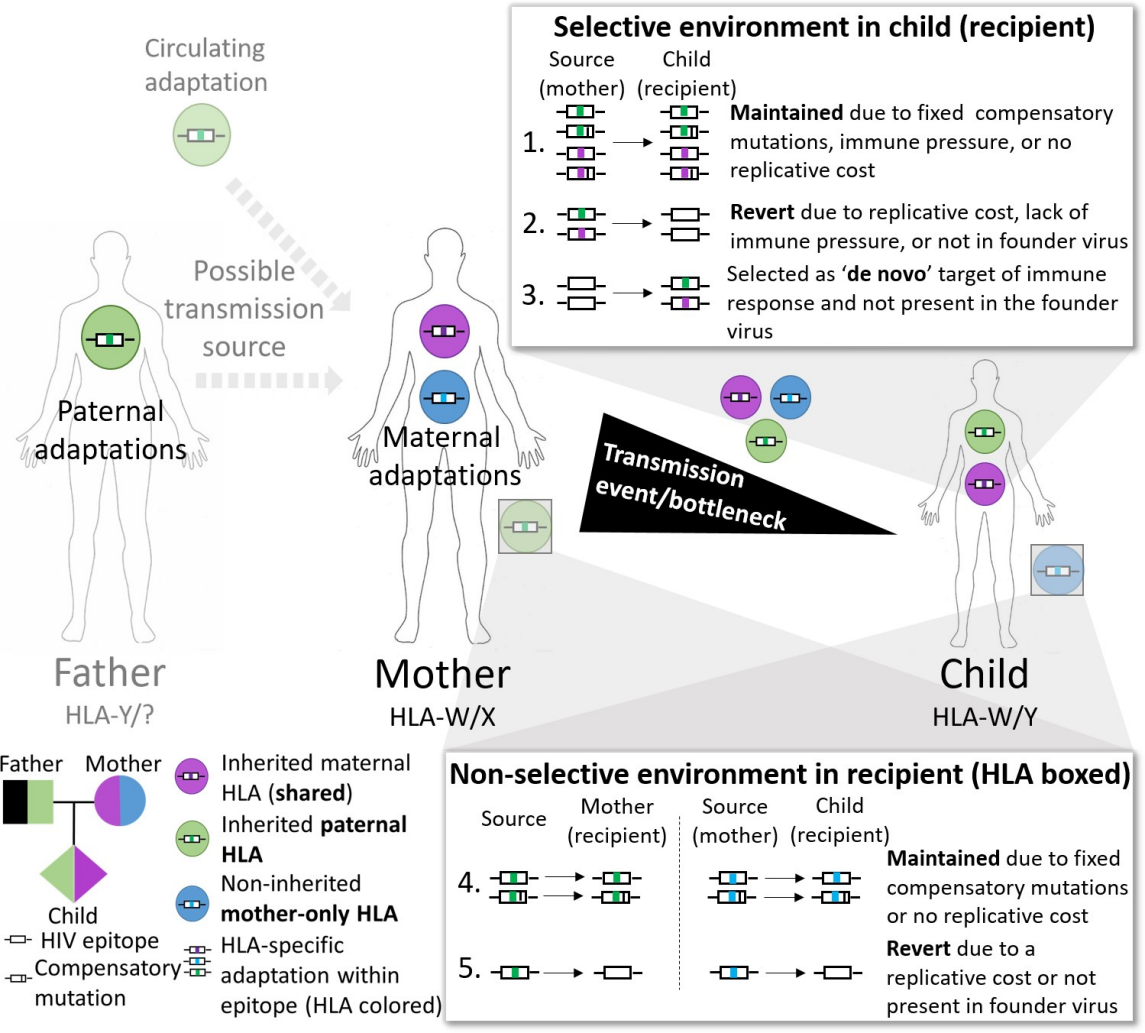
Adaptations can be transmitted, but their maintenance or reversion is dependent on the immune selection environment of the recipient and replicative cost. In this cohort the original source of HIV in the mother is unknown and is depicted as either the child’s father or another individual (represented by circulating adaptation). In this model, the original source virus adapts to the immune selection environment in the mother before being transmitted to the child. While in the mother, HIV adaptations corresponding to the child’s paternal HLA allele are in a non-selective immune environment. Such adaptations may revert (scenario 5) to the non-adapted form due to a replicative cost or may be maintained (scenario 4) if the adaptation has no replicative cost or has linked compensatory mutations. *De novo* adaptations corresponding to the maternal HLA alleles may arise in the mother’s autologous virus due to immune selection pressure. During transmission of the virus from the mother to the child there is a bottleneck event with the resultant founder viruses in the child likely carrying HIV adaptations relevant to both maternal and paternal HLA alleles. The maintenance (scenario 1 or 4), reversion (scenario 2 or 5), and selection of *de novo* adaptations (scenario 3) in the child’s virus will be dependent on the new immune selection environment and/or replicative cost of the adaptations.

HIV adaptations that impose a cost to the replicative capacity of the virus are expected to revert to the non-adapted form upon transmission to an HLA-mismatched individual, as the site is likely no longer under immune selection pressure. This is supported by data showing that transmission of an adapted virus to an HLA-mismatched individual correlates with lower viral load and better disease outcome [9, 10]. However, viral adaptations with low/no cost to replicative capacity or those that are fully compensated by secondary mutations may not revert, becoming fixed within circulating viruses and accumulating in the consensus sequence of a host population over time [4, 11] (see model in Fig 1).

The high heterozygosity observed in HLA genotypes means that horizontal HIV transmission events usually occur in fully or partially HLA-mismatched donor/recipient pairs. As such, horizontally transmitted HIV adaptations that are incidentally relevant to the recipient’s HLA alleles are likely to represent fixed or near fixed adaptations in the circulating virus of the population. These adaptations likely incur minimal replicative cost or have accumulated linked compensatory mutations over time such that the genetic barrier is ‘too high’ for the virus to revert to the non-adapted form [7]. In vertical HIV transmissions, presuming no selection bias at transmission, the level of adaptation within the transmitted virus is likely to be higher due to the sharing of at least 50% of HLA alleles between mother and child. This level of pre-adaptation in the transmitted virus may be further increased to encompass 100% of the child’s HLA alleles if the source virus has passaged from the father of the child to the mother [12]. These features of vertical transmission allow the evaluation of the replicative cost of specific HIV adaptations in the context of matched and mismatched HLA environments, as well as determine whether new HIV T cell epitopes may emerge in heavily pre-adapted strains. These observations are important as T cell responses against HIV are detectable in the first few days of infection in children [13, 14]. Others have also suggested that a large number of immunodominant T cell epitopes have already adapted in the mother, leaving the child to target subdominant epitopes in a less effective immune response [15, 16].

The study of HIV adaptation in vertical transmission events has the potential to inform prophylactic vaccine design in combatting circulating strains, or for a therapeutic vaccine given that latent reservoirs are likely to harbor HLA-adapted viruses. This study utilised archived samples from a historical cohort of mother/child pairs with HIV clade B infection to examine the intra- and inter-host dynamics of HIV adaptations following transmission into HLA-matched and -mismatched environments using deep sequencing.

## Results

### Confirmation of vertical HIV transmission

**Fig 2:**
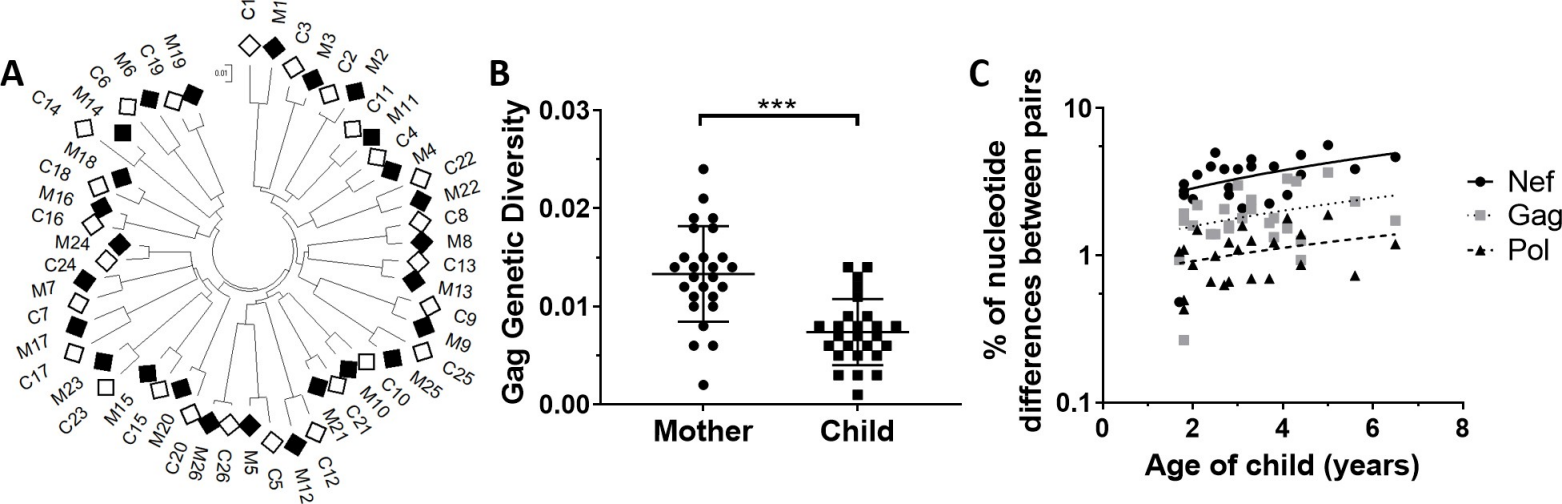
(A) Maximum likelihood phylogenetic tree of Gag majority sequences confirms mother-to-child transmission (mother, M = closed diamond; child, C = open diamond) with no evidence of larger transmission networks within cohort (see S1B and S1C Fig for Pol and Nef). (B) Intra-individual genetic diversity of Gag quasispecies was significantly higher in the mother than the child (N *=* 26, p = 0.0001; paired t-test) with similar results obtained for Pol and Nef (see S1E and S1F Fig). Data are represented as mean ± SE. (C) Significant positive correlation across all proteins for nucleotide differences (%) between mother/child pairs and age at sampling (time since transmission) (N = 23, p = 0.007; mixed-effects linear regression model). There was a significant difference between the nucleotide differences (%) for the different proteins (N = 23, p<0.0001; linear regression). p<0.001 (***).

Samples from 26 mother/child pairs (Table 1) were utilised to obtain deep sequencing data for the HIV proteins Gag, Pol and Nef. Phylogenetic analysis of the protein sequences (using the majority amino acid at each position) for all three proteins strongly supported HIV transmission from mother-to-child, with no evidence to suggest larger transmission networks within the cohort (Fig 2A and S1A–S1C Fig). Genetic diversity of the quasispecies in all three proteins, calculated for each individual, was significantly higher in the mother than the child (Gag p = 0.0001, Pol p = 0.003, Nef p = 0.007; paired t-test; Fig 2B and S1D–S1F Fig), supporting a bottleneck event at transmission. Gag genetic diversity was associated with viral load in the mother (p<0.001; and for Pol p = 0.23, Nef p = 0.79; linear regression), but not the child (p = 0.20; and for Pol p = 0.43, Nef p = 0.55). Furthermore, genetic diversity was not correlated with the number of starting HIV copies in the mother or child (Gag p = 0.67, Pol p = 0.72, Nef p = 0.93 for the mother, and Gag p = 0.32, Pol p = 0.99, Nef p = 0.72 for the child; linear regression), nor treatment status in the child (Gag p = 0.60, Pol p = 0.99, Nef p = 0.25; ANOVA).

**Table 1:** Subject information and clinical data of mother/child pairs.

Subject ID	Sex	Age^Ϯ^ (y)	ART	Time on ART^Ϯ^ (m)	HLA-A		HLA-B		HLA-C		Viral load (log copies/ml)^^^	CD4%^#^
**Mother 1**		45.3	AZT+3TC+NVP	5.5	3303	7401	1516	5703	1402	0701	1.70	13.3
**Child 1**	M	4.1			3303	2902	1516	1302	1402	0602	4.13	47.8
**Mother 2**		25.6	d4T+3TC+ABC	30.4	8001	3002	1801	1503	0202	0210	2.08	27.9
**Child 2**	M	4.4			8001	7401	1801	5201	0202	1601	5.23	54.8
**Mother 3**		23.9			0202	3002	5101	3501	0401	1601	4.22	17
**Child 3**	F	4.4	DDI+3TC+NVP	14.6	0202	0202	5101	5301	0401	1601	4.41	4.9
**Mother 4**					0301	3601	4403	5301	0401	0401	4.92	34.1
**Child 4**	M	1.8	AZT+3TC+NVP	16.3	0301	6802	4403	1510	0401	0304	1.70	44.9
**Mother 5**		32.8			6802	3402	4201	5301	0401	1701	3.33	25.6
**Child 5**	F	2.8			6802	0101	4201	5301	0401	1701	4.81	47.3
**Mother 6**		39.7			0101	6801	0801	5703	0701	1801	1.70	29.7
**Child 6**	F	4.3	AZT+3TC+NVP	17.0	0101	3303	0801	1516	0701	1402	4.02	10.2
**Mother 7**		36.0			3001	0201	0702	3901	1505	0702	5.97	29.2
**Child 7**	M	3.3			3001	3402	0702	3501	1505	0401	5.09	42.2
**Mother 8**		39.2			7401	6802	1510	5301	0304	0401	4.56	14.9
**Child 8**	M	3.8	AZT+3TC+NVP	12.8	7401	6801	1510	0702	0304	0702	3.19	57.3
**Mother 9**		32.4			6602	7401	5801	1503	0701	0210	4.20	35.9
**Child 9**	F	2.4			6602	3002	5801	1402	0701	0802	4.61	49.6
**Mother 10**		40.9			2902	0201	4403	0702	1601	0702	4.95	46.6
**Child 10**	M	3.1			2902	3601	4403	5301	1601	0401	4.96	26.2
**Mother 11**		32.8			7401	7401	5301	1503	0701	0210	5.48	13
**Child 11**	F	3.8	AZT+3TC+NVP	7.8	7401	0301	5301	0702	0701	0702	3.55	42.1
**Mother 12**		34.8			6802	2402	0702	4901	0702	0701	5.79	5.4
**Child 12**	F	5.6			6802	3402	0702	4201	0702	1701	4.68	18
**Mother 13**		25.7			0201	7401	3501	0702	1601	0702	2.51	39.9
**Child 13**	F	4.1	AZT+3TC+NVP	13.9	0201	0201	3501	4402	1601	0501	5.12	38.9
**Mother 14**		25.7			3303	3002	7801	0702	1601	0702	1.70	28.2
**Child 14**	M	3.0	DDI+3TC+NVP	7.3	3303	2301	7801	5703	1601	1801	5.14	2.2
**Mother 15**		29.0			3002	6802	1402	0702	0802	0702	3.77	24
**Child 15**	F	3.7			3002	7401	1402	3501	0802	0401	5.06	37
**Mother 16**		22.8			0202	0201	4202	5301	0401	1701	3.73	19.6
**Child 16**	F	1.7	AZT+3TC+NVP	17.8	0202	3001	4202	5301	0401	1701	4.36	17.3
**Mother 17**		36.6			2301	0201	1402	3910	0802	1203	5.24	17.27
**Child 17**	F	5.0	AZT+3TC+NVP	22.2	2301	0301	1402	4901	0802	0701	4.33	31.6
**Mother 18**		35.5			3001	3002	4201	5801	1701	0302	3.65	43.3
**Child 18**	F	6.5			3001	6801	4201	5802	1701	0602	3.06	29.8
**Mother 19**		35.7			3301	2902	7801	4901	0701	1601	5.47	54.3
**Child 19**	M	2.8			3301	0101	7801	5701	0701	1601	5.41	19.3
**Mother 20**		31.1			0301	3001	3501	4201	0701	1701	5.06	20.9
**Child 20**	M	2.0			0301	3301	3501	1516	0701	1402	4.21	21.8
**Mother 21**		31.8			6801	0201	4016	4501	0802	1601	4.44	28
**Child 21**	F	2.5			6801	3002	4016	0801	0802	0701	4.75	26.3
**Mother 22**		28.1			3402	3002	4403	5801	0304	0701	5.16	43
**Child 22**	M	3.3			3402	7401	4403	5301	0304	0701	3.80	37.2
**Mother 23**					3001	0202	8101	4201	1801	1701	4.75	41
**Child 23**	M	1.8			3001	7401	8101	4901	1801	0701	1.70	40.1
**Mother 24**		33.6			2402	6802	4403	5301	0401	0401	4.46	52.9
**Child 24**	M	2.1	AZT+3TC+NVP	15.3	2402	6601	4403	4101	0401	0701	5.46	50.9
**Mother 25**		41.6			0201	7401	5702	4501	1801	1601	5.09	16.6
**Child 25**	F	2.7			0201	3303	5702	1516	1801	1402	4.26	50.1
**Mother 26**		26.6			7401*	6802	5301	1510	0401	0301	unknown	24.51
**Child 26**	M	1.8	AZT+3TC+NVP	1.0	7401*	7401*	5301	1503	0401	0202	unknown	36.06

ϮTime from sampling

^Median (interquartile range; IQR) time from sampling 0 (0–2) months for mothers, 2 (0–6) months for children

#Median (IQR) time from sampling 6 (2–7) months for mothers, 2 (0–6) months for children

*HLA completed to 4-digit resolution; ART = antiretroviral therapy; Drug abbreviations: AZT (zidovudine), NVP (nevirapine), 3TC (lamivudine), DDI (didanosine), ABC (abacavir), d4T (stavudine); F = female; M = male; m = months; y = years. Proviral HIV sequencing was performed for subjects M1, B4, M6, C8, M14, and M19 with the remaining HIV sequencing from plasma.

Genetic variation between the majority HIV nucleotide sequences for each mother/child pair was significantly correlated with time since transmission (taken as age of child at sampling; p = 0.007; mixed-effects linear regression; Fig 2C), with similar trends observed for all three proteins. Nef exhibited the greatest genetic difference between the sequences of the mother/child pairs (mean ± standard error (SE) = 3.44% ± 0.24%), followed by Gag (1.87% ± 0.15%) and then Pol (1.05% ± 0.08%). There was no effect on genetic variation between mother and child when the child’s treatment status was considered (Gag p = 0.55, Pol p = 0.66, Nef p = 0.25; ANOVA). These results confirmed the continual diversification of HIV strains after the bottleneck event that is directly proportional to time since divergence (transmission) and reflects the known structural and functional constraints of the proteins [17, 18].

### Most HIV adaptations in the mother’s autologous virus are fixed or near fixed resulting in transmission of highly adapted virus to the child

We initially identified known HIV adaptations [19], defined as specific amino acids at sites across the HIV genome associated with host carriage of specific HLA alleles, in the mother’s viral quasispecies as representative of the source virus at the time of transmission. Of the HIV adaptations present in the mother’s quasispecies (and relevant to the HLA alleles in the mother/child pair) 74% were fixed (at 100% frequency in the quasispecies) with most other adaptations near-fixed at ≥90% frequency in the quasispecies (S2 Fig). These results support the likely transmission to the child of most adaptations present in the mother, irrespective of a bottleneck event.

**Fig 3:**
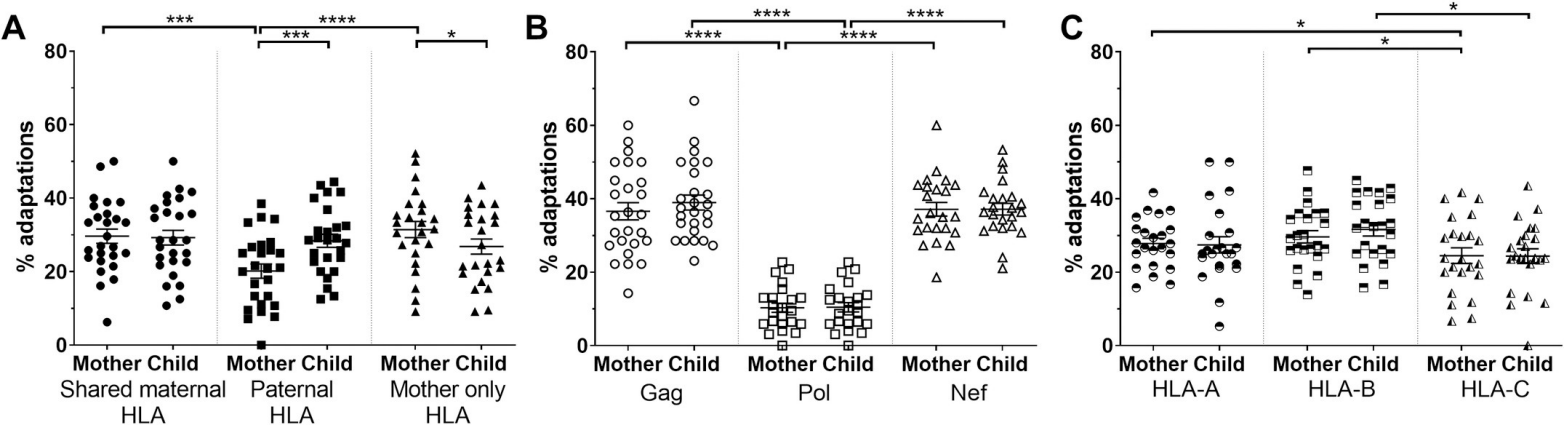
Autologous virus is adapted in both mother and child. The proportion of known HIV adaptations observed in the autologous virus of mother/child pairs. (A) Adaptations were separated into three groups: 1) shared maternal HLA alleles present in both mother and child; 2) paternal HLA alleles only present in the child; and 3) mother-only HLA alleles present only in the mother. (B) Adaptations based on location within HIV proteins Gag, Pol, and Nef. (C) Adaptations sorted by HLA locus. All analyses were performed using mixed-effects logistic regression incorporating HLA inheritance, HIV protein, and HLA locus. Data are represented as mean ± SE. N *=* 23, p<0.0001 (****), p<0.001 (***), p<0.01 (**), p<0.05 (*).

HIV quasispecies were highly adapted with on average 27.5% and 28.2% of known adaptations present relevant to the HLA allele repertoire in the mother and child, respectively (see S1 Table for the number of adaptations examined for each subject). We then classified HIV adaptations in the autologous virus in the child according to whether they were relevant to the HLA allele inherited from the mother (shared maternal HLA allele), inherited from the father (paternal HLA allele), or the maternal HLA allele not passed to the child (mother-only HLA allele). In the child’s viral quasispecies, there was no significant difference in the proportion of HIV adaptations when separated into these three HLA allele categories (Fig 3A). However, in the mother’s quasispecies there was a significantly higher proportion of HIV adaptations relevant to the HLA alleles she carried (shared maternal and mother-only HLA alleles) than the paternal HLA allele she does not carry (p<0.001; mixed-effects logistic regression incorporating HLA inheritance, HIV protein, and HLA locus; Fig 3A). There was no difference in the proportion of HIV adaptations relevant to the two maternal HLA alleles (p = 0.4). This was as expected given the virus in the mother would be under immune selection pressure from the HLA alleles she carries and not at sites that would come under immune selection pressure due to the paternal HLA allele she does not carry.

HIV adaptations were more prevalent in Gag and Nef compared to Pol in both the mother and child (p<0.0001) and occurred in similar proportions in Gag and Nef (p = 0.5 and p = 0.5 for the mother and child, respectively; Fig 3B). There was also a modestly higher prevalence of HIV adaptations relevant to the HLA-B than -C alleles (p = 0.04 and p = 0.02 for the mother and child, respectively), and HLA-A than -C alleles in the mother (p = 0.03), but no difference between HLA-A and -B alleles (p = 0.8; Fig 3C).

When comparing the different proportions of HIV adaptations in the mother and child, there was no difference in the level of adaptation relevant to the shared maternal HLA allele (p = 0.7; Fig 3A). However, in the child’s HIV sequence there was a marginal reduction in adaptations relevant to the mother’s HLA allele not inherited by the child (mother-only HLA) (p = 0.05), and a significant increase in the level of adaptation relevant to the paternal HLA allele in the child that was not present in the mother (p = 0.0006). These differences in the proportion of HIV adaptations in the mother and child are likely to reflect the maintenance or reversion of transmitted adaptations and the *de novo* formation of adaptations; factors that are dependent on the immune selection environment of the recipient and replicative cost of the adaptation as depicted in the model presented in Fig 1.

### HIV adaptation following mother-to-child transmission can reflect in vivo replicative cost of specific HIV adaptations

**Fig 4:**
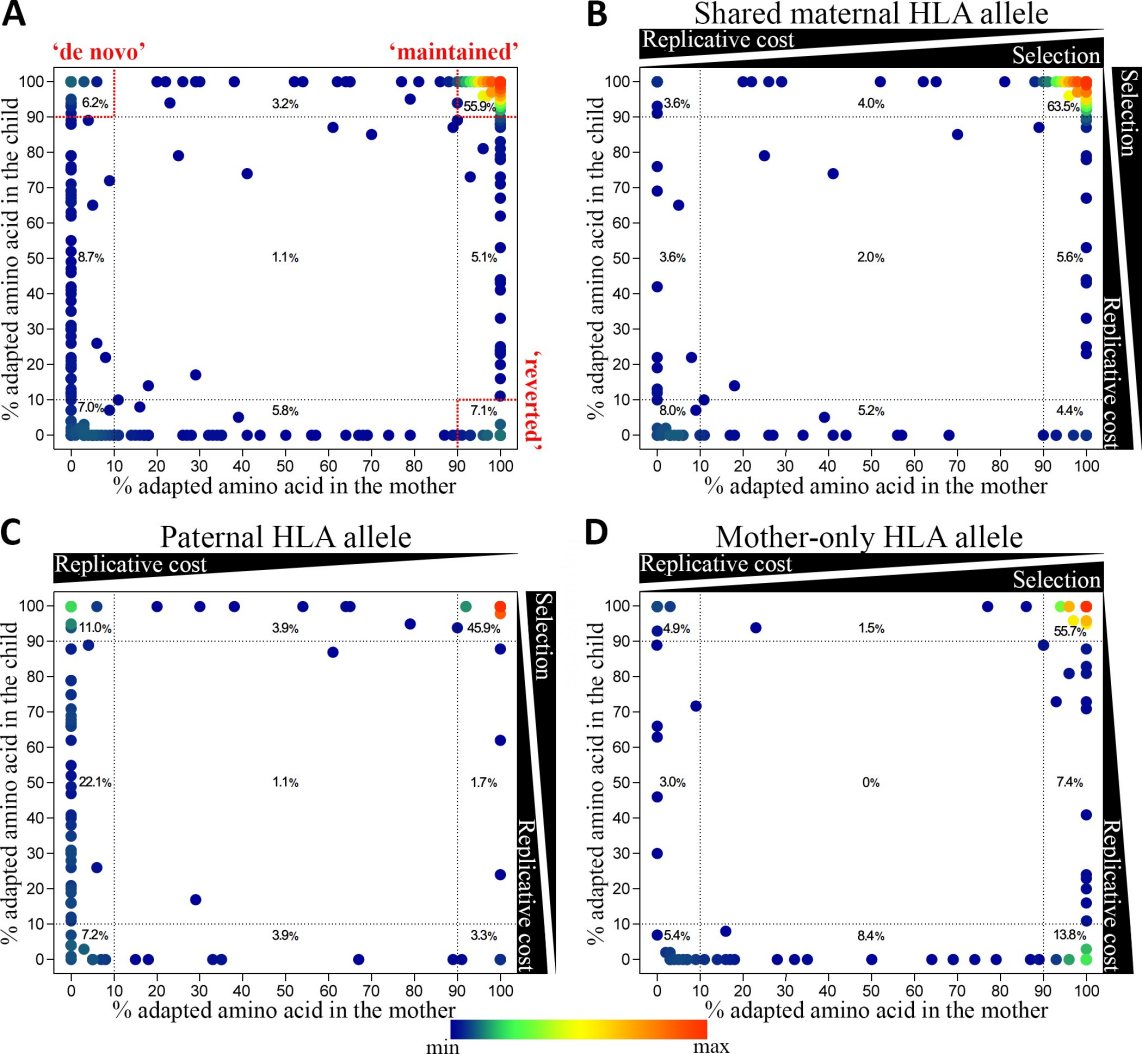
Adaptation dynamics following transmission affected by bottleneck event, immune selection pressure, replicative cost and drift. (A) The % of quasispecies for all adapted amino acids in Gag, Pol, and Nef in the mother/child pairs (N *=* 633) were plotted on the x-axis for the mother, and the y-axis for the child. Points are superimposed on each other and the color range reflects number of superimposed points with red (maximum, max) and blue (minimum, min). ‘Maintained’ adaptations are present in the top right corner (≥90% in the child), ‘reverted’ in the bottom right corner (<10% in the child), and ‘*de novo*’ adaptations along the y-axis (<10% in the mother), with respect to the child. (B) Adaptations that are potentially under immune selection pressure in both the mother and child (shared maternal HLA; N *=* 249; scenarios 1–3 in the child from Fig 1). (C) Adaptations that are in a non-selective immune environment in the mother, but potential selective environment in the child (paternal HLA; N = 181; scenarios 4–5 for the mother and 1–3 for the child from Fig 1). (D) Adaptations that are potentially under a selective environment in the mother, but a non-selective immune environment in the child (mother-only HLA; N = 203; scenarios 1–3 for the mother and 4–5 for the child from Fig 1). In all panels, percentages denote adaptations that are present in that quadrant for that grouping. Panels B-D also indicate the likely influence of selection on, and replicative cost of (theoretical), adaptations along the axes.

To assess the impact of immune pressure on transmitted adaptations, we again separated adaptations into those relevant to the shared maternal HLA allele (immune selective environment in both mother and child), paternal HLA allele (immune selective environment only in the child) and mother-only HLA allele (immune selective environment only in the mother). Adaptations were defined as fixed if the amino acid was present in the quasispecies at 100% (i.e. the only amino acid present at this position), and near-fixed if they were ≥90%. Examination of all HIV adaptations observed in the mother and/or child (633 adaptations overall) showed 68.1% were present at or close to fixation (≥90%) in the mother’s quasispecies (Fig 4A). Restricting the analysis to only these fixed or near-fixed adaptations in the mother (≥90%) shows those relevant to either the paternal or shared maternal HLA alleles were largely maintained in the same frequency range (≥90%) in the child (paternal: 90.2%, shared maternal: 86.4%, p = 0.3 for difference; mixed-effects logistic regression). This was significantly more than the proportion observed at a frequency ≥90% in the child for sites relevant to the mother’s HLA allele not inherited by the child (mother’s only: 72.4%, p = 0.005 vs paternal, p = 0.009 vs shared maternal; Fig 4B–4D). Notably, at the sites relevant to the mother’s HLA allele not inherited by the child, nearly 18% of the adaptations observed at a frequency ≥90% in the mother’s quasispecies were at a frequency of less than 10% in the child, compared to approximately 6% of adaptations lost at sites relevant for inherited HLA alleles (Fig 4A). This indicates that although reversion of adaptations occurs once transmitted into a non-selective HLA environment, many adaptations are retained.

To further explore the possibility of a larger transmission network involving the child’s father, we calculated and compared circulating adaptation scores (representing adaptation pre-existing in majority circulating sequences) and individual adaptation scores of the transmitted virus (mother’s HIV sequence) for each HLA allele present in the child (maternally and paternally inherited). Across maternally inherited HLA-A and -B alleles, the level of HIV adaptation in the transmitted virus was significantly higher than the level of HIV adaptation in the circulating virus (p<0.0001; mixed-effects linear regression; S3 Fig). However, this effect was not shown for paternally inherited HLA alleles (p = 0.4). As time since infection was unknown in the mother, it may be that HIV adaptations restricted by the paternally inherited HLA allele that incurred a replicative cost reverted within the mother and the remaining transmitted adaptations held little replicative cost, as supported by a lower level of reversion for transmitted adaptations relevant for the paternally inherited HLA allele (Fig 4C), explaining the lower level of adaptation to the paternal HLA alleles in the mother’s HIV sequence.

The frequency distribution of the corresponding adaptations in the HIV quasispecies of the mother/child pairs also highlighted a dearth of adaptations occurring between 10–90% (Fig 4A). This was also observed when comparing the frequency of all amino acids in Gag, Pol, and Nef in the mother/child pairs (S4A Fig). This pattern may reflect the reduction in diversity observed after a transmission event in which other non-immune-based selection pressures (including replicative cost), and drift may be relevant.

Examination of the different HIV proteins shows the largest variation in adaptation frequencies occurred in Nef, with 83.9% of adaptations in Pol occurring at a frequency of ≥90% in both the mother and child (S4B–S4D Fig). These data again reflect the known differences in substitution rates (due to functional and structural constraints) for the different HIV proteins.

### Known immunodominant HIV CD8^+^ T cell epitope follows expected reversion/maintenance pattern following transmission

To highlight a specific HIV CD8^+^ T cell epitope for which there was both reversion and maintenance of adaptation following transmission, we examined the immunodominant T242N adaptation for the HLA-B*57/58-restricted Gag epitope TW10, which has a known replicative cost and linked compensatory mutations [20–22]. Within the cohort there were eight mother/child pairs where at least one subject carried the HLA-B*57/58 allele (Table 2). In three of the pairs, only the mother had HLA-B*57/58 and the T242N adaptation reverted in the child in all cases where the adaptation was present in the mother (non-selective immune environment in the child). In the three pairs where both mother and child had HLA-B*57/58 (selective environment for both), all mothers had the T242N adaptation, with two children maintaining the adaptation. Furthermore, in the two cases where the child had HLA-B*57/58 but the mother did not (virus passing from a non-selective immune environment in the mother to a selective immune environment in the child), the T242N adaptation was not present in the mother, but was present in the child (at 100%). In another study using samples from this cohort, child 19 (*de novo* adaptation in Table 2) was shown to have an interferon gamma (IFN-γ) T cell response to TW10 [22] confirming immune selection pressure. In concordance with our results, this study also described linked compensatory mutations offsetting replicative cost in all subjects with the T242N adaptation [22].

**Table 2:** Presence of the T242N adaptation in the HLA-B*57/58-restricted Gag T cell epitope TW10.

		Epitope Sequence										Compensatory Mutation (Position no.)				
		TW10 (Gag 240-249)										218A	219Q	223V	228I/L	248A
		T	S	**T**	L	Q	E	Q	I	G	W					
Presence of HLA-B*57/58 in mother and child (predict maintenance)	Mother 9	.	.	N	.	.	.	.	.	A	.	P	.	.	M	G
	Child 9	.	.	N	.	.	.	.	.	A	.	P	.	.	M	.
	Mother 18	.	.	N	.	.	.	.	.	A	.	V	H	.	.	.
	Child 18	.	.	.	.	.	.	.	.	A	.	V	.	.	M	.
	Mother 25	.	.	N	.	.	.	.	.	.	.	V	H	.	M	G
	Child 25	.	.	N	.	.	.	.	.	.	.	V	H	.	.	G
Presence of HLA-B*57/58 in mother only (predict reversion)	Mother 1	.	.	N	.	.	.	.	V	A	.	V	.	.	.	.
	Child 1*	.	.	.	.	.	.	.	.	A	.	V	.	.	P	.
	Mother 6	.	.	N	.	.	.	.	.	A	.	V	.	.	.	.
	Child 6*	.	.	.	.	.	.	.	.	T	.	.	.	.	.	T
	Mother 22	.	.	.	T	.	.	.	.	.	.	V	H	.	M	G/D
	Child 22*	.	.	.	.	.	.	.	.	D	.	V	H	.	M	D
Presence of HLA-B*57/58 in child only (predict *de novo*)	Mother 14*	.	.	.	.	.	.	.	.	.	.	V	H	N/I	M	G
	Child 14	.	.	N	.	.	.	.	.	A	.	V	.	P	.	.
	Mother 19*	.	.	.	.	.	.	.	.	A	.	V	H	N/I	.	.
	Child 19	.	.	N	.	.	.	.	.	A	.	V	H	I	.	.

Position for known adaptation in bold

*Subject does not have B*57/58;. = identity; Compensatory mutations as described in [22, 23].

### Lack of correlation between described replicative capacity scores and transmitted adaptations likely reflects extensive network of compensatory mutations

To better understand the maintenance and reversion of the transmitted adaptations, specific variations were correlated to previously described replicative capacity scores across the HIV genome [24] (Table 3). Three amino acid sites were of particular interest given multiple subjects harbored the same adaptations: Pol 12, Gag 146 and Nef 83. Pol T12K has been described as having a lethal replicative capacity score, yet out of the six mothers who carried this adaptation, the adaptation was maintained in all children after transmission. Of note, two out of the six children who maintained the adaptation did not possess the restricting HLA allele. Interestingly, scores that reflect the capacity for variability at sites across the HIV genome based on autologous HIV sequences in a host HIV-infected population (HIVpC; [25]) and deeper evolutionary time (including SIV orthologous from different species; EvC) (see methods) better reflect the outcomes from transmission (Table 3; positive HIVpC and EvC scores likely reflect sites that can accommodate variability while negative scores reflect conservation). Similarly, for Gag A146P the adaptation was maintained (3/5 children that did not carry the relevant HLA allele) even though it had a predicted attenuated replicative capacity. In contrast, the HIVpC and EvC scores suggested less constraint. The Nef A83G mutation had a replicative capacity score indicative of tolerance. Nine mothers carried this adaptation, yet this adaptation reverted in five of the corresponding children (adaptation was present at ≥90% in the mother), while the other four maintained the adaptation. Overall, there was no obvious correlation between the maintenance or reversion of transmitted adaptations as observed in the mother/child data and the previously described replicative capacity phenotypes based on a single or limited subset of mutations on the same genetic backbone [24]. Furthermore, these data suggest compensatory mutations and other linked variants greatly influence the replicative capacity of these adaptations; relationships that will be captured in this study.

**Table 3:** Replicative capacity and associated conservation scores for HIV adaptations observed in this study.

Replicative capacity	Protein	Missense substitution	Number of mother/ child pairs	Maintained (n; S,M,P)	Reverted (n; S,M,P)	*De novo* (n; S,M,P)	HIV population conservation (HIVpC)	Evolutionary conservation (EvC)	Site-specific constraint prediction	BLOSUM62 score	HLA restriction	Associated epitope (HLA restriction)
Lethal	Gag	N385S	1	0	0	1 (0,1,0)	0.806	3.624	V	1	B*15	
		P487T	1	1 (1,0,0)	0	0	1.819	0.372	V	-1	B*07	
		T490K	2	2 (1,0,1)	0	0	1.822	-0.535	V	-1	A*68:01	
	Pol	T12K	7	6 (3,2,1)	0	1 (0,1,0)	4.087	1.001	V	-1	A*30	
		K14R	1	1 (1,0,0)	0	0	0.263	0.268	V	2	B*51:01	
		D559E	1	0	1 (0,0,1)	0	1.954	0.378	V	2	B*42:01	
Attenuated	Gag	A146P	5	5 (1,3,1)	0	0	1.823	1.389	V	-1	B*39:01, B*57:03, C*08	
		S173T	2	2 (0,2,0)	0	0	1.079	0.299	V	1	B*57:03	
		A374T	1	1 (0,1,0)	0	0	4.007	1.822	V	0	B*45:01	AEAMSQVTNS (B*45:01)
		L463F	2	2 (1,1,0)	0	0	0.243	1.768	V	0	A*29:02	
	Pol	Q433H	1	1 (1,0,0)	0	0	-0.234	-0.357	C	0	C*07	
Tolerated	Gag	R91K	1	0	0	1 (0,0,1)	4.007	0.92	V	2	C*06:02	
		I147L	2	2 (0,0,2)	0	0	1.822	1.677	V	2	B*13:02, B*57:01	ISPRTLNAW (B*57:01)
		R286K	2	2 (1,0,1)	0	0	1.822	-0.106	V	2	B*14, B*52, C*08:02	
		G357S	1	0	1 (0,1,0)	0	1.911	0.893	V	0	B*07:02	GPGHKARVL (B*07:02)
	Nef	Y81F	3	1 (1,0,0)	1 (0,1,0)	1 (1,0,0)	0.085	1.222	V	3	B*35:01	VPLRPMTY (B*35:01)
		A83G	11	4 (3,1,0)	5 (2,3,0)	2 (0,1,1)	0.917	3.109	V	0	A*03:01, B*07, B*57:03, B*58:01, C*03, C*07:02	RPMTYKAAL (B*07:02); KAAFDLSFF (B*57:03/B*58:01); AALDLSHFL (C*03)
		D86N	1	1 (1,0,0)	0	0	-0.455	-0.879	C	1	C*08:02	AAVDLSHFL (C*08:02)
		E93D	1	1 (1,0,0)	0	0	-0.564	-0.711	C	2	B*44, C*16:01	
		K94N	1	1 (1,0,0)	0	0	-0.687	-0.696	C	0	B*08:01	FLKEKGGL (B*08:01)
		I114V	9	7 (4,0,3)	1 (1,0,0)	1 (0,0,1)	0.389	-0.495	V	3	A*02:02, B*07:02, B*35, C*06:02, C*07, C*14	KRQEILDLWVY (C*07)
		Y115H	2	1 (1,0,0)	0	1 (0,0,1)	-0.668	0.037	V	2	C*07:01, C*14:02	KRQEILDLWVY (C*07)
		Y120F	4	2 (2,0,0)	2 (0,2,0)	0	-0.091	1.42	V	3	A*30:02. C*14:02	
		Q125H	1	0	1 (0,0,1)	0	-0.342	-0.745	C	0	C*06:02	YFPDWQNYT (C*06)
		D151E	1	0	0	1 (1,0,0)	1.454	-0.153	V	2	A*02:01	** **
		V153I	1	0	0	1 (0,1,0)	-0.174	-0.502	C	3	A*02	
		P176T	3	3 (1,2,0)	0	0	0.705	-0.062	V	-1	B*53:01	
		R188L	2	1 (0,1,0)	1 (0,0,1)	0	1.155	1.549	V	-2	A*74:01, C*07:02	

C = conserved site (HIVpC & EvC < 0); P = paternal HLA allele; S = shared maternal HLA allele; M = mother-only HLA allele; V = variable site (HIVpC & EvC > 0); Lethal (<0.1), Attenuated (0.1–0.2), Tolerated (>0.2) as described in [24].

### *De novo* HIV adaptations occur in the child after transmission

**Fig 5:**
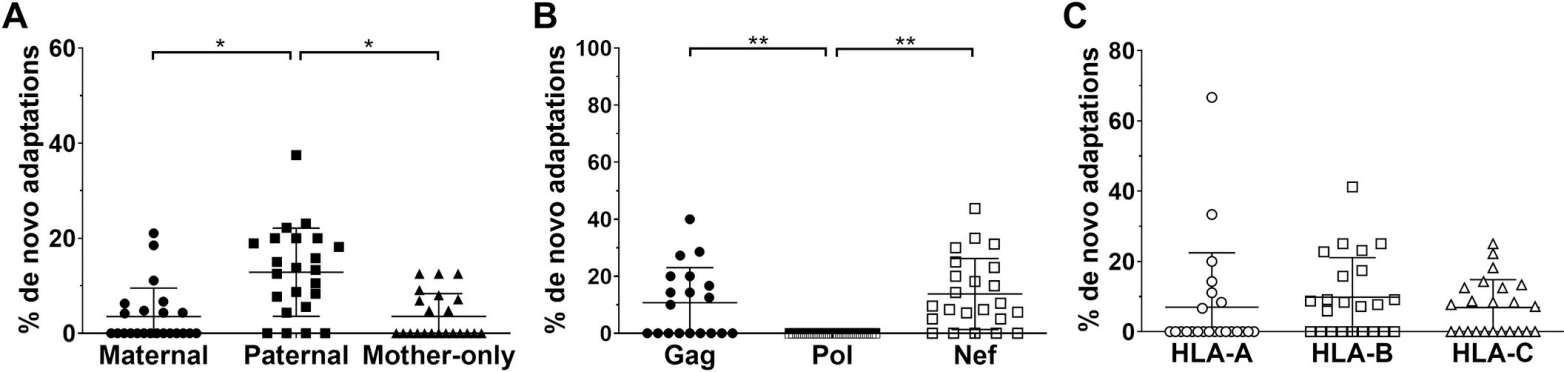
*De novo* HIV adaptations occur in the child following transmission. (A) Significantly higher proportion of *de novo* adaptations were relevant to the paternal HLA allele than both maternal HLA alleles. (B) Gag and Nef had significantly more *de novo* adaptations relative to Pol. (C) There was no significant difference between *de novo* adaptations relevant to the different HLA loci. All analyses were performed as in Fig 4. N *=* 23, p<0.01 (**), p<0.05 (*). Data are represented as mean ± SE.

We found several adaptations had become fixed (or near fixed; ≥90%) at the time of sampling in the child for amino acids that were either not present, or at low frequencies (<10%), in the mother (6.2% of adapted sites overall; Fig 4A). Fixation in the child was more likely to occur at sites relevant to paternally inherited HLA alleles compared to sites relevant to the mother’s HLA alleles (odds ratio, OR [95% confidence interval, CI] = 2.4 [1.3–4.6], p = 0.008; mixed-effects logistic regression; Fig 4B–4D and Fig 5A). These amino acids may represent *de novo* adaptations due to immune selection pressure in the child and likely reflect the larger number of adaptation sites available relevant to the paternal HLA allele that do not show evidence of the adapted amino acid in the transmitted virus (p = 0.002; paired t-test, S5 Fig). There was no difference in the proportion of *de novo* adaptations between relevant shared maternal HLA alleles and mother-only HLA alleles (p = 0.4; mixed-effects logistic regression; Fig 4B and 4D). Furthermore, there was no difference in *de novo* adaptation and treatment status in the child (p = 0.67; t-test).

Consistent with other results, a higher proportion of *de novo* adaptations occurred in Nef and Gag compared to Pol (p<0.002 for both; Fig 5B). However, there was no significant difference between HLA loci (p>0.1; Fig 5C). Overall, 41% of *de novo* adaptations were fixed in the child, however there was no significant correlation between *de novo* adaptation and age of the child at sampling. Intracellular cytokine staining (ICS) was used to confirm *de novo* responses in 13 children (an example shown in S6 Fig with the peptide pool tested listed in S2 Table).

### Adaptation scores correlated with clinical measures of disease outcome in the mother, but not in the child

**Fig 6:**
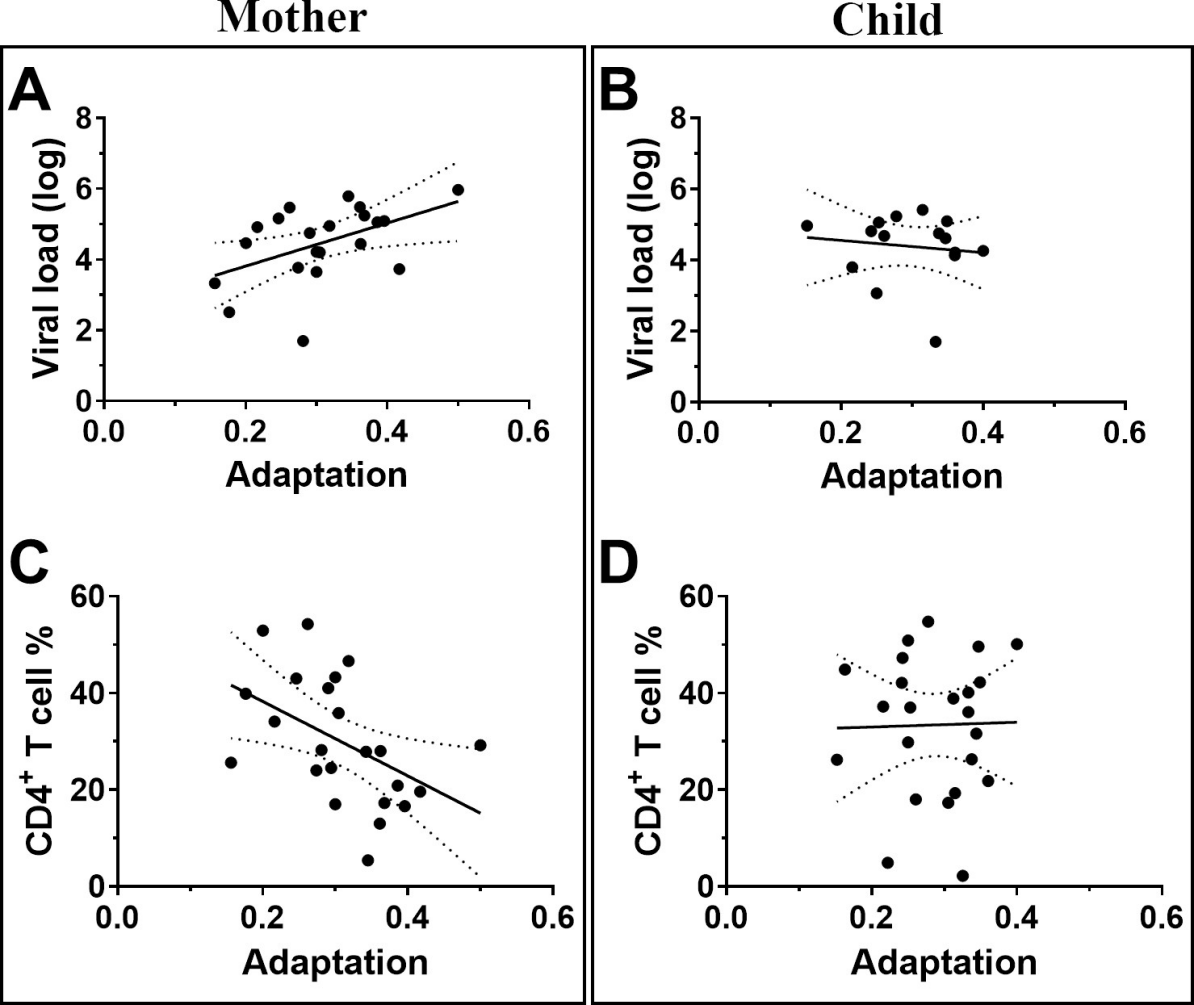
Adaptation scores are correlated with clinical measures of disease outcome in the mother, but not the child. In the mother, adaptation scores positively correlated with viral load (N = 21, Spearman’s r = 0.44, p = 0.05; A), and negatively correlated with CD4^+^ T cell % (N = 23, Spearman’s r = −0.50, p = 0.02; C). In the child, there was no correlation between adaptation scores and viral load (N = 14, p = 0.7; B) or CD4^+^ T cell % (N = 23, p = 0.9; D). 95% confidence intervals are shown.

Total adaptation scores of the mother’s autologous virus positively correlated with viral load (N *=* 21, Spearman’s r = 0.44, p = 0.05; Fig 6A), and negatively correlated with CD4^+^ T cell % (N *=* 23, Spearman’s r = −0.50, p = 0.02; Fig 6C), consistent with prior research [3]. In children, we found no correlation between adaptation scores and viral load (N *=* 14, p = 0.7; Fig 6B), CD4^+^ T cell % (N *=* 23, p = 0.9; Fig 6D) or treatment status (N = 23, p = 0.52). Furthermore, there was no correlation between *de novo* adaptation and disease outcome (p>0.6).

### Transmission of adapted virus to the child influences selection of HIV T cell targets

**Fig 7:**
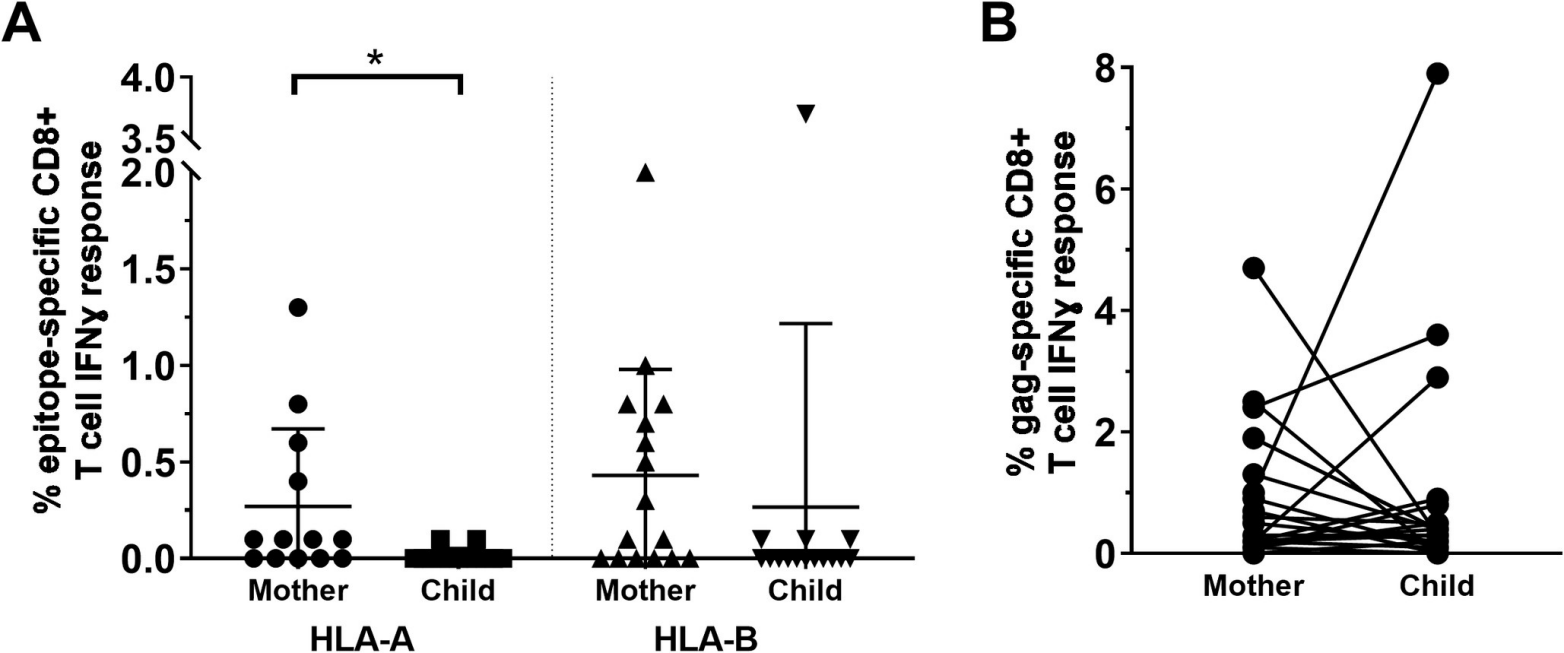
HIV-infected children responded to a broad array of Gag peptides but had a limited response to optimal CD8^+^ T cell epitopes, including immunodominant T cell epitopes. (A) There was a significantly higher response in the mother than the child for HLA-A-restricted optimal CD8^+^ T cell epitopes (N = 13, p = 0.03; Wilcoxon signed-rank test) and a trend for HLA-B-restricted optimal CD8^+^ T cell epitopes (N = 15, p = 0.08). Data are represented as mean ± SE. (B) There was no difference in IFN-γ responses to a Gag peptide pool in the mother and child (N = 24, p>0.9).

To better understand the influence of a transmitted pre-adapted virus on the immune targets of the child, we measured IFN-γ responses in 17 mother/child pairs following stimulation with optimal HIV CD8^+^ T cell epitopes (including immunodominant T cell epitopes) in Gag, Pol, and Nef (http://www.hiv.lanl.gov/content/immunology; Fig 7A). All children except one had low or no response to the optimal T cell epitopes. In contrast, the mothers had a significantly greater IFN-γ response to the same peptides for HLA-A (N *=* 13, p = 0.03; Wilcoxon signed-rank test), but only a slightly higher IFN-γ response for HLA-B (N *=* 15, p = 0.08) even though there was no significant difference in the number of variants to the optimal peptides in the autologous virus of the mother and child. Of note, on average about 55% of the tested optimal peptides had at least one amino acid change relative to the mother’s autologous virus. Of these peptides, approximately 84% were also different in the child (S3 Table).

We then extended this analysis to include an expansive Gag peptide pool (includingnumerous peptides across the protein incorporating known epitopes and variants) tested in 24 mother/child pairs. For the Gag peptide pool, IFN-γ responses were present in 83% of mothers and 75% of children with no significant difference in the magnitude of IFN-γ responses (p>0.9; Fig 7B). Furthermore, IFN-γ responses to the Gag peptide pool did not correlate with age in the mother or child. For the mother, there was a trend for a negative correlation between anti-Gag IFN-γ T cell responses and viral load (N *=* 23, Spearman’s r = −0.35, p = 0.1), but not in the child (p = 0.4). These data indicate that adaptation of consensus HIV-1 T cell epitopes restricted by maternal HLA alleles is likely related to lack of recognition in children, but children maintain the ability to generate de novo immune responses.

An additional factor contributing to the variation in the anti-HIV T cell responses observed between mother and child pairs could be due to differences in the endoplasmic reticulum aminopeptidase 1 (*ERAP1*) allotypes (S4 Table). *ERAP1* allotypes are known to influence peptide trimming [26] and consequently have been shown to affect the peptide repertoire presented to T cells [27] and immunodominance [28]. Three mother/child pairs were of particular interest (pairs 9, 14 and 22) due to likely differences in trimming ability between mother and child (i.e. one subject had predicted hypoactive only trimming allotypes versus prototype/wildtype form). There was no obvious difference in reversion and maintenance patterns or in abilities of T cells to respond to peptides in these pairs relative to others; albeit there were only a small number of mother/child pairs with likely discordant trimming efficiencies.

## Discussion

### High-resolution sequencing enhances the ability to assess dynamics of viral adaptation following transmission

Transmission of HLA-associated HIV adaptations [7, 9] have predominantly been analysed using Sanger-based sequencing methods that typically identify one dominant viral strain. As such, the effects of selective pressures on multiple or emerging quasispecies are not evident in these studies. Here we utilised deep sequencing to assess the dynamics of reversion or fixation of specific adaptations within host quasispecies in the context of both selective and non-selective immune pressures. We observed no impact of treatment status in the child on HIV diversity, genetic distance, measures of disease outcome or adaptations. Although 11 children were on treatment, seven of these showed NRTI and NNRTI drug resistance mutations and it is also possible that adherence could be an issue, as indicated by unsuppressed viral loads.

Overall, the data showed that most adaptations were fixed (100%), or near fixed (≥90%), in the mother’s quasispecies and therefore most likely transmitted, with a bias towards those adaptations relevant to her own HLA repertoire. Most of these adaptations were maintained in the child (at ≥90%), reflecting the adaptive benefit of these changes for shared HLA-restricted immune responses (same immune selection environment; Fig 1 scenario 1).

Some adaptations reverted following transmission (Fig 4). These observations likely reflect the range of replicative costs associated with these adaptations, as the mother is the proximate source of the virus for the child and the transmitted adaptations should reflect the cost/benefit balance within the maternal selective environment. This is supported by the reversion of the T242N adaptation in the well-described Gag HLA-B*57/58-restricted T cell epitope TW10 in children who do not possess the restricting HLA (Fig 1 scenario 5). In contrast, transmitted HIV adaptations relevant to the paternal HLA alleles were lower in number, but also less prone to reversion in the child. These adaptations likely have low replicative cost or are linked with compensatory mutations as they have been passed through the mother (in a non-selective immune environment) from either the father or other hosts within the population and likely would have reverted before transmission to the child if there was a high replicative cost (Fig 1 scenario 4). We were unable to differentiate between these two possibilities in this cohort as the donor source of HIV to the mothers was either not known or not recorded. Loss of adaptation at sites relevant to the mother’s HLA allele that the child did not inherit likely reflects ‘reversion’ in a non-selective immune environment (Fig 1 scenario 5). Reversion or maintenance of transmitted adaptations is dependent on drift (particularly early after transmission), replicative cost and immune selection pressure. The presence of linked compensatory mutations offsets replicative cost and may partially explain the limited amount of reversion observed in this dataset. This is also supported by the lack of concordance between published replicative capacity phenotype and outcome of a set of the adaptations after transmission. Scores based on levels of variability observed in autologous HIV sequences from a large population-based cohort (HIVpC) and in SIV/HIV sequences representing a deeper evolutionary scale (EvC) provide a better prediction (albeit this is only for the few sites carried by multiple subjects) of whether adaptations are likely to be maintained or reverted. This may be indicative of a more complex dynamic encompassing compensatory mutations that would be difficult to detect using single site mutations. Exploring replicative capacity in an in vivo setting such as described in this study highlights the need for further characterisation of these potential compensatory mutations. The limited reversion of transmitted adaptations in mother/child pairs has been previously described for HIV clade C using Sanger-based sequencing [15, 18]. In addition, lack of reversions may lead to a higher prevalence of circulating adaptations at a host population level, impacting disease progression of newly infected individuals [11, 29, 30].

The data also show that a significant proportion of the adaptations fell within Gag and Nef, two of the most immunogenic HIV proteins [31] and support other studies of the transmission of HIV adaptations [12, 18, 32]. Interestingly, although the overall genetic variation in Nef was greater than Gag in the mother/child pairs, the proportion of HIV adaptations between the two proteins was not significantly different. Contrastingly, a similar study using Sanger-based sequencing of mother/child pairs found a higher density of mutations in Nef compared to Gag and Pol, with Gag higher than Pol [18]. Furthermore, population-based studies have also found a higher density of HLA-associated HIV polymorphisms in Nef than Gag or Pol [32].

While others have observed higher HLA-B-restricted HIV-specific CD8^+^ T cell responses than for HLA-A and -C [33], and there are known associations between certain HLA-B alleles and elite control of HIV infection [34], we found only a modest difference in the proportion of adaptations relevant to the different HLA loci with a higher proportion of adaptations relevant to HLA-B alleles than -C, but not -A.

### Level of HIV adaptation correlates with maternal outcome but not the child

Transmission of pre-adapted HIV has been associated with worse disease outcome in horizontal transmissions, due to the potential for the virus to undermine the recipient’s immune response [3, 35]. The results of this study were congruent with previous research in adults [3], but the same correlation was not found in children and others have suggested that HIV behaves differently in children than adults [36–38]. However, a mitigating factor to correlating disease outcome and adaptation in this cohort is the possible survivor bias in the children as most children were older than two years of age. Previous reports have shown that >50% of ART-naïve children die by age 2 [39] and that anti-HIV immune responses early in life may differ [40]. Accordingly, the data from this cohort may not represent the full clinical spectrum anticipated from a cohort sampled earlier post-transmission. Furthermore, infants (≤ 24 months) who could be classified as severely immunosuppressed (CD4^+^ T cell % ≤ 15) have been shown to exert a decreased immune selection pressure on HIV, associated with worse clinical outcomes [41]. As there are only three children in this cohort with a CD4^+^ T cell % less than 15%, it is suggestive that there is a survivor bias in this cohort and these children, who had survived past 2 years, were potentially capable of exerting a greater anti-HIV response.

### Transmission of pre-adapted virus influences selection of immune targets in the recipient

In vertical transmissions, it has previously been suggested that a large number of T cell targets required for an effective HIV-specific T cell response in the child have already adapted in the mother [15, 16]. However, the child can still mount an immune response to HIV and is capable of exerting immune pressure on the virus [12, 42], as supported in this study by the child’s ability to target Gag and produce *de novo* adaptations. The T cell epitopes targeted by the child may not be the same immunodominant targets ‘seen’ by the mother. The remaining T cell targets may be subdominant, potentially affecting the child’s ability to mount an effective anti-HIV T cell response and hence contributing to the reduced immune control observed in children [3, 12]. Previously, HLA-B*57/58 positive children who inherited the T242N adaptation for the immunodominant TW10 Gag epitope from HLA-B*57/58 positive mothers have shown no response to TW10, whereas those children with HLA-B*57/58 negative mothers did show a response [22]. Therefore, children may not be capable of recognising and mounting a response to immunodominant T cell epitopes if the transmitted strain is pre-adapted.

Here we show that when both mother and child are stimulated with peptides representing optimal CD8^+^ T cell epitopes (many of which are immunodominant) there is clearly a lack of a response in the child relative to the mother. This is consistent with acutely infected adults where there is a diminished capacity to respond to epitopes that are already adapted, as measured by IFN-γ responses [3, 6]. For a large proportion of the peptides tested, the autologous virus in the mother (and child) did not match the peptide sequence. This difference in response between mother/child pairs is not present when the stimulating peptides include a more expansive array of peptides (Gag peptide pool) that encompass a broader range of T cell epitopes. However, we observed a negative correlation between the magnitude of the IFN-γ response to the Gag peptide pool and viral load in the mother (as shown for other HIV-infected adults [43]), but not in the child. This could reflect the reduced repertoire of T cell epitopes available to target in the child infected with a highly pre-adapted virus leading to the targeting of subdominant epitopes, or it may be that these particular epitopes are both replicatively neutral or do not contribute significantly to immune control. As subdominant epitopes are suggested targets of T-cell vaccines [44] it is vital to understand how adaptation within these epitopes would affect the efficacy of a vaccine. Furthermore, variation in T cell responses in mother/child pairs may reflect differences in *ERAP1* allotypes that result in differences in the peptide repertoire presented to T cells [26] between mother and child. We identified only three mother/child pairs with likely differences in peptide trimming efficiency but were unable to confirm any differences in these pairs. However, the small number of pairs with likely different phenotype may reflect an added burden for children in vertical HIV transmissions given the inheritance of the *ERAP1* allotypes such that the peptide trimming efficiency of mother and child may often be the same, limiting the ability of the child’s immune response to ‘see’ other T cell epitopes.

Among the *de novo* adaptations observed in the child’s virus in this study, there was a bias towards those relevant to paternal HLA alleles. We speculate that in those cases where HIV was transmitted through the father and then the mother, adaptations may have reverted in the mother due to replicative cost and then re-selected in the child as *de novo* adaptations. Our assignment of *de novo* adaptations in these cases used the most parsimonious approach. Although the majority of *de novo* adaptations were for HLA alleles present in the child, a small number were for mother only HLA alleles (not inherited by the child). It is possible that this may be due to overlapping epitopes restricted by other HLA class I or class II alleles. As there was no correlation between adaptation in the child and clinical outcomes, we hypothesized a potential impact of *de novo* adaptations on outcome, although we were unable to observe a correlation between *de novo* adaptations and viral load in the child.

Treatment status of the children is not likely to have affected our ability to detect anti-HIV T cell responses as these responses have been shown to occur in utero, and persist, with the capability of clonal expansion in vivo, suggestive of functionality [40, 45]. In this study, children on ART had a median age (IQR) of ART initiation of 2.7 (1.3–3) years (the youngest was 9.5 months).

Pediatric HIV infection has been of particular interest for understanding mechanisms of immune control and as a potentially more tractable pathway to HIV cure, owing to the ability to act in very early infection, the tolerogenic immune environment in infants, and potential to reduce reservoir size early. However, there has been research into determining the ability of the infant’s immune system to exert an anti-HIV response. Understanding this response is critical to the development of vaccines for children with, or susceptible to, HIV infection from their mothers.

The implications of transmission dynamics on the goal of cure strategies has been uncertain. Here we have examined the impact of HIV adaptation and show that, as in other settings, HLA exerts a dominant selective force on HIV. Pediatric hosts are commonly infected with viruses that have adapted to shared maternal HLA. Furthermore, HIV derived from a transmission chain from paternal to maternal hosts may result in a relatively rapid pathway to adaptation to the complete HLA repertoire of the child, though this is modulated by the costs to replicative capacity of individual adaptations. Despite the high rate of pre-adaptation, there was clear evidence of anti-HIV T cell responses against subdominant epitopes within children, showing that they are capable of exerting an immune selection pressure on the virus. High-resolution sequence analysis revealed the intra-host reversion and maintenance dynamics of transmitted adaptations in different immune selection environments, which acts as an estimate of the replicative cost and immune benefit of specific adaptations *in vivo*. It is hoped a better understanding of these dynamics can be used to inform vaccine design to maximise replicative cost of adaptations within the host and to avoid the selection of adaptations that incur minimal replicative cost.

## Materials and methods

### Study subjects

Plasma, peripheral blood mononuclear cells (PBMCs), and clinical data were obtained from 26 clade B HIV-1 infected mother/child transmission pairs who were recruited through the GHESKIO Program in Port-au-Prince, Haiti. Data from three mother/child pairs have previously been reported: pairs 6, 19, and 25 [22]. CD4^+^ T cell % values were obtained from flow cytometry experiments on thawed cryopreserved PBMCs for 49 of the 52 subjects. For the three subjects without PBMCs available, CD4^+^ T cell % values from the clinical record were used as there was a strong correlation between CD4^+^ T cell % values obtained from flow cytometric analyses of cryopreserved PBMCs and clinical data (clinical data was at the same time point as PBMCs or within 6 months; p<0.0001; linear regression; S7 Fig). Furthermore, comparison of CD4^+^ T cell % values in HIV-positive and negative subjects in six laboratories found a significant positive correlation (p<0.0001) between fresh and cryopreserved PBMC samples [46].

The median age (interquartile range; IQR) in years at time of sampling for the mothers (N *=* 24; two unknown) was 32.8 (27.7−36.1) and children (N *=* 26) 3.2 (2.4−4.1). The median viral load (IQR) in log copies/mL for the mothers (N *=* 25) was 4.46 (3.65−5.09) and children (N *=* 25) 4.41 (4.02−5.06). The median CD4^+^ T cell % (IQR) for the mothers (N *=* 26) was 22.45 (15.94−29.58) and children (N *=* 26) 29.94 (21.85−39.65). No difference in viral load or CD4^+^ T cell % between children and treatment status was observed (p = 0.55 and p = 0.34, respectively; t-test). This result was supported by the analysis of protease-reverse transcriptase sequences for the 11 children on treatment that showed seven had mutations likely to confer high-level resistance to at least two of the three drugs in their treatment regime according to the Stanford University HIV Drug Resistance Database (https://hivdb.stanford.edu/).

The duration of HIV infection in the mother and exact mode of HIV transmission to the child was not known, but was most likely *in utero*, peripartum or during breast-feeding. A summary of the clinical and genotyping data for all individuals is shown in Table 1.

### Ethics statement

Subjects were recruited through the GHESKIO Program in Port-au-Prince, Haiti. All subjects and/or legal guardians signed written informed consent prior to participation and samples were anonymized. Institutional review board (IRB) approval for sample collection was obtained prior to the commencement of the original study (IRB 0409007436/0504-294; Cornell University). Subsequent ethics approval was obtained for the use of archived material in the repository at Vanderbilt University Medical Center (IRB 100061).

### DNA and viral RNA extraction

Viral RNA was isolated from 200μL of plasma using the MagMAX-96 Viral RNA Isolation Kit (Life Technologies, CA, USA) and genomic DNA was extracted from 200μL of PBMCs using the QIAGEN Extraction Kit (QIAGEN, NRW, Germany), as per the manufacturer’s instructions.

### HLA genotyping

High-resolution HLA class I (HLA A; B; C) and class II (HLA DQA1; DQB1; DRB1; DPB1) typing was performed by an American Society for Histocompatibility and Immunogenetics (ASHI) and National Association of Testing Authorities (NATA) accredited laboratory at the Institute for Immunology and Infectious Diseases, Murdoch University, Western Australia using locus-specific PCR amplification of genomic DNA. The assay was adapted from a previously published barcoded-PCR protocol [47] with modifications to the primer sequences (See S5 Table). Briefly, RNA was amplified by 12 PCR reactions using subject specific barcoded primers. Amplified products were quantified, normalised, and pooled by subject. Libraries were created for sequencing and quantified using the Kapa Universal qPCR Library Quantification Kit (Kapa Biosystems Inc., MA, USA), as per the manufacturer’s instructions. Samples were sequenced on an Illumina MiSeq using a 2×300 paired-end chemistry kit (Illumina Inc., CA, USA), as per the manufacturer’s instructions. Reads were quality-filtered and passed through a proprietary allele calling algorithm and analysis pipeline using the latest IMGT HLA allele database [48]. Where PBMCs were not available, HLA class I typing previously completed at DCI Tissue Typing Laboratory (TN, USA) was used, with 2-digit HLA typing completed to 4-digit where necessary using an online HLA completion tool available at https://phylod.research.microsoft.com [3].

### *ERAP* genotyping

High-resolution *ERAP1* and *ERAP2* genotyping was performed at the Institute for Immunology and Infectious Diseases, Murdoch University, Western Australia using uniquely indexed locus specific PCR amplification of genomic DNA. Seventeen single nucleotide polymorphisms (SNPs) for *ERAP1* and two SNPs for *ERAP2* were assayed: rs3734016, rs73148308, rs26653, rs26618, rs27895, rs2287987, rs27434, intron, rs73144471, rs27529, rs78649652, rs30187, rs10050860, rs111363347, rs17482078, rs27044, rs375081137, rs2248374 and rs2549782. Positive controls were DNA from either lymphoblastoid K562 (donated by Dr Frans Claas, Leiden University Medical Centre, Netherlands) or C1R [49] cells lines with known *ERAP* genotypes with sterile water as a negative control. PCR products were pooled and purified using Agencourt AMPure XP (Beckman Coulter, CA, USA) with libraries created for sequencing using TruSeq adapters (Illumina Inc.) and quantified using the KAPA Universal qPCR Library Quantification Kit (Kapa Biosystems Inc., MA, USA), as per the manufacturer’s instructions. Samples were sequenced on an Illumina MiSeq using a 2×300bp paired-end chemistry kit (Illumina Inc.), as per the manufacturer’s instructions. Reads were quality-filtered and aligned to the reference sequence *ERAP1* or *ERAP2* in CLCbio Genomics Workbench 11 (QIAGEN Bioinformatics). Alignment files were exported in BAM format and analysed for coverage and SNPs using visual genomics analysis studio (VGAS), an in-house program for visualising and analysing sequencing data (http://www.iiid.com.au/software/vgas) [50]. *ERAP1* allotypes were assigned based on their trimming activity (wildtype/prototype, hyperactive or hypoactive) according to five SNPs as described previously [26]; M349V, K528R, D575N, R725Q and Q730E.

### Copy number determination

Viral and proviral HIV copy numbers were determined using RT-qPCR and qPCR, respectively. Viral RNA samples were tested using the GoTaq Probe RT-qPCR System (Promega Corporation, WI, USA), and proviral samples tested using the iTaq Universal Probes Supermix (Bio-Rad Laboratories, CA, USA), as per the manufacturer’s instructions. See S6 Table for qPCR cycling conditions and S7 Table for the primer sequences. HIV sequences were custom synthesised into gBlocks (Integrated DNA Technologies, IA, USA) for use as a standard at an initial concentration of 1×10^8^ copies followed by seven 10-fold dilutions. Standard curves had an R^2^ ≥ 0.997 and an efficiency of 99.7%– 103.3%. There was no significant difference between the copies of viral RNA used for the initial RT-PCR in mother and child samples (p = 0.35; paired t-test). Viral load was not associated with the number of copies in the initial RT-PCR (p = 0.18; linear regression).

### Viral sequencing and analysis

HIV Gag, Pol, and Nef sequences were amplified by nested PCR (following RT-PCR for viral RNA samples). RT-PCR used 4μL of sample with the SuperScript III One-Step RT-PCR System with Platinum Taq High Fidelty Kit (Life Technologies) and proviral sequencing used Platinum *Taq* DNA Polymerase High Fidelity Kit (ThermoFisher, MA, USA), as per the manufacturer’s instructions. See S6 Table for PCR cycling conditions and S7 Table for primer combinations and amplicon lengths.

### Next-generation sequencing (NGS)

PCR amplicons were pooled for each subject and enzymatically fragmented using the NEBNext dsDNA Fragmentase kit where required (New England Biolabs, MA, USA), as per the manufacturer's instructions, with an average fragment length of 500bp (range of 400-1000bp). Fragments were purified and used as input into library prep with unique indexes, as per the manufacturer’s instructions (Kapa Biosystems Inc.). The Kapa Universal qPCR Library Quantitation Kit (Kapa Biosystems Inc.) was used to quantify the libraries prior to sequencing on the MiSeq sequencer using a 2×300 paired-end chemistry kit (Illumina Inc.), as per the manufacturer’s instructions. Briefly, raw sequencing reads were quality trimmed using the standard MiSeq parameters (Q score of 15 and adaptor trimmed) and mapped to a partial reference sequence HXB2 in CLCbio Genomics Workbench 11 (QIAGEN Bioinformatics), accession number PRJNA525760. Alignment files were exported in BAM format and imported into VGAS for further coverage and SNP analysis. Reports, consensus, and majority sequences were constructed using a 3% nucleotide cut off. The median of each subject’s average coverage across Gag, Pol and Nef was 38,165 (IQR 30443–43632). There was no difference in sequencing coverage (calculated per copy sequenced–as described above) between mother and child across all proteins (Gag p = 0.96; Pol p = 0.99; Nef p = 0.97; ANOVA). See S8 Table for sequencing summary information, including reads per copy.

### Confirmation of variant frequency using primer IDs

The use of large size amplicons to cover much of the HIV genome, as described in this study, precludes the use of unique primer identifiers (PIDs) to directly quantitate RNA species as previously described [51, 52]. However, in order to determine if the approach described here identified variants at a frequency that correlates to the original RNA species population (no PCR bias), five subjects were selected for PID analysis using subject-specific primers for a smaller amplicon in Nef. Nef was selected due to its higher diversity and location of adaptations. The initial cDNA conversion used a primer that contained a unique molecular identifier (8mer), a barcode (subject specific) and a non-specific region to allow PCR amplification attached to a gene specific primer. An upper limit of 20,000 RNA copies was utilized to allow each cDNA template to contain a unique PID sequence. cDNA was purified twice to remove any unbound PIDs before PCR amplification with a sample specific primer and a generic primer for the nonspecific region using PrimeSTAR GXL DNA Polymerase (Takara Bio Inc., Japan). First round products were purified using the MinElute PCR Purification kit (QIAGEN), as per the manufacturer’s instructions. Samples then underwent a second round PCR using a nested sample specific and generic primer before purification and NGS, as above. See S6 Table for PCR cycling conditions and S7 Table for primer combinations. Twenty thousand reads were sampled and used to determine the majority consensus of each unique PID. A PID was only included if it was present in four or more sequences. For comparison the same protocol was performed using the same sample but without PID.

Analysis of the frequency of sites within the HIV amplicon sequences with or without primer IDs for five subjects showed a significant correlation (all sites p<0.0001 r = 0.86; only sites with variation from 100% p<0.0001 and r = 0.75; linear regression; S8 Fig). At 614 (91%) sites the same amino acid was at 100% in both sequences, with the majority amino acid in the remaining 61 sites the same for all sites except nine, where in one instance the majority amino acid in the primer ID sequence was at 55% compared to 48%. In the remaining eight sites, the amino acid was present above 10% (our cut-off for adaptation analysis) in the sequence without primer ID. Furthermore, the primer ID sequence showed good concordance with the corresponding patient data set. For example, in one subject 10/11 sites with variation had a correlation of p<0.0001, with one amino acid, although present, having a higher frequency than the primer ID sequence. All other majority amino acids were concordant.

### Mean genetic distance

To calculate mean genetic distance (MEGA version 7) [53], 5000 full length reads were reconstructed by assigning the amino acid(s) at each position while maintaining the ratio observed in the full coverage (e.g. if a position had 50% A and 50% G, A and G were randomly assigned to each of the 5000 reads at this position with a final 50:50 ratio of A:G). The best model function was used to analyse a representative sequence sample to identify the Tamura-Nei model (G = 0.34) as the best-fit model for the data. A second method was used where aligned sequence files for each subject were constructed by reiteratively mapping reads (mean of 211bp) back onto the reference sequence HXB2 such that there was no overlap between reads. The first 5000 concatenated files were used to determine mean genetic distance. The length of the reads meant that on average only 7 reads were required to cover the length of Gag, allowing for some linkage of variations across each protein. Comparison of these two methods showed a correlation of p<0.0001 and r = 0.59 (linear regression).

### Phylogenetic analysis

To confirm mother-child HIV transmission, a phylogenetic tree was constructed. The best model function was used to analyse representative sequences for Gag to identify the Hasegawa-Kishino-Yano model (G = 0.72) with the maximum likelihood method in MEGA version 7.0 [53]. HIV subtype B was confirmed using the REGA HIV subtyping tool [54].

### Identification of HLA-associated HIV adaptations and analysis

Previously determined HLA-associated HIV adaptations were used in analyses [19]. These adaptations represent statistically significant associations between individual amino acids in the HIV genome and a specific HLA class I allele. For each Gag, Pol, and Nef alignment, a list of amino acids was produced using VGAS and compared to a list of previously determined HLA-associated polymorphisms to determine adaptation scores for each subject; where adaptation scores quantify the degree of adaptation of an HIV quasispecies population to a specific HLA repertoire [3].

Adaptations present at ≥10% frequency of the quasispecies population were considered biologically relevant based on our clinical experience with low frequency drug resistance mutations (M. John personal communication). However, when this cut-off was increased by increments of 5% to fixation (100%), most correlations remained significant. Adaptations were also divided based on the inheritance of the restricting HLA; maternal (present in the child and inherited from the mother), paternal (present in the child, but not the mother, and inherited from the father), and mother-only (present in the mother, but not inherited by the child).

Three adaptation scores were determined using methods similar to those previously described [11, 18, 19, 36]: 1) adaptation; 2) transmitted adaptation; and 3) circulating adaptation. Our adaptation scores correlated with scores produced using an online tool developed by Carlson et al. (https://phylod.research.microsoft.com/; p<0.001; S9 Fig) [3]. A total number of five HLA-associated polymorphisms were required for inclusion in the scoring system.

The score for ‘adaptation’ reflects the total adaptation of HIV quasispecies within the host to their HLA repertoire. This value was calculated as the ratio of the number of adaptations present, to the number of adaptations possible for the individual’s HLA alleles. The score for ‘transmitted adaptation’ was only calculated for the child and reflects the level of adaptation of the transmitted virus to the child’s HLA alleles. It was calculated as the ratio of the number of adaptations present in the mother’s HIV sequence (transmitted virus), to the number of adaptations possible for the child’s HLA alleles. For this analysis, adaptations shared with the mother were assumed to have been transmitted (if present ≥10% in the mother), and adaptations not shared with the mother were assumed to be *de novo* adaptations in the child (if present ≥10%). The score for ‘circulating adaptation’ was calculated for each HLA allele present in the cohort and was created for each sequence (mother’s sequences only), providing the mother and child do not possess the specific HLA allele. The average was taken to give the circulating adaptation score for an individual HLA allele.

### Replicative capacity

Replicative capacity for adaptations present in mother/child pairs were determined from previously published data [24]. There were three HIV replicative capacity classifications: lethal, attenuated, and tolerated. Lethal adaptations have a score of ≤0.1 and have a significant impact on replicative capacity, attenuated adaptations have a score of 0.1–0.2 and have a moderate impact on replicative capacity, and tolerated adaptations have a score of ≥0.2 and have little to no impact on replicative capacity. These mutations were, for the most part individually, incorporated into a pNL4-3 backbone.

### Conservation scores

HIV population amino acid and site-specific evolutionary conservation scores for full-length HIV-1 subtype M Gag, Pol and Nef proteins were computed using the ConSeq web server [55]. For HIV population conservation (HIVpC) scores, sequences from the AIDS Clinical Trial Group (ACTG) were utilised in the HIV population analysis [25]. Simian immunodeficiency virus (SIV) evolutionary conservation (EvC) scores were determined using sequences from the public database GenBank. Evolutionary conservation scores were calculated relative to all associated SIV orthologues using maximum likelihood phylogenetic analyses. Single site evolutionary rate was estimated from the phylogeny, adjusted for varying numbers of protein homologues and protein length. BLOSUM scores for the adaptations were calculated by means of the BLOSUM62 substitution matrix [56].

### Individual peptide design

We designed peptides for 13 of the mother/child pairs according to *de novo* adaptations identified in the child. HIV sequences containing the *de novo* adaptation (between 8–11 amino acids) were screened for potential T cell epitopes using the online programs NetMHC 4.0 and NetMHCpan 4.0 (http://www.cbs.dtu.dk/services/). Peptides corresponding to known T cell epitopes in the HIV LANL database (http://www.hiv.lanl.gov/content/immunology) and/or having the strongest predicted binding results from the programs were selected and synthesised (GenScript Biotech Corp., NJ, USA). Peptides corresponding to the wild type (transmitted from the mother) and variant (*de novo* in the child) epitopes were obtained.

### Intracellular cytokine staining (ICS)

Cryopreserved cells were thawed and pulsed with a Gag peptide pool (peptides from AIDS Reagent and Repository at a final concentration of 1μg/mL per peptide, catalogue number 11554), HLA-specific optimal epitope pools from the HIV database at www.lanl.gov, and customised peptide pools containing both the wild type and variant epitopes overnight to examine T cell responses in both the mother and child. SEB (Merk Millipore, VT, USA) was used as a positive control at 1μg/mL. Briefly, approximately 1-2×10^6^ PBMCs/well were incubated for 4 hours at 37°C and 5% CO_2_ with co-stimulatory molecules CD28 and CD49d (BD Biosciences) before a 2-hour stimulation with a specific peptide pool. Brefeldin A (BD Biosciences) was added prior to an overnight incubation at 37°C and 5% CO_2_. Culture medium was RPMI supplemented with 10% fetal calf serum, 1% penicillin streptomycin, 10mM glutamine and 10mM HEPES. Cells were washed and stained with the monoclonal antibodies anti-CD8-APC A750 (Life Technologies), anti-CD3-A700, anti-PD1-BV421, anti-CD14/19-V500, and anti-CD4-PcPCy5.5 (BD Biosciences), fixed and permeabilized before a second wash and staining with intracellular antigens IFN-γ-FITC, CD69-APC, and CD137-PE (BD Biosciences, NJ, USA). Live/Dead Fixable Aqua (V500; Life Technologies) and CD-14/19 were used to exclude dead and undesired cells. Samples were visualised using a 4-Laser Fortessa (BD Biosciences) and analysed with BD FACSDiva software (BD Bioscience) and FlowJo. A response was deemed to be positive if it was three times above the media response and ≥50,000 events were recorded. ICS using peptide stimulation was utilised as previous research has suggested this is a more accurate representation of the functional T cell response in adults and children than using tetramers or ELISpot [57], with differing responses observed between tetramer and ELISpot [58].

### Statistical analysis

Parametric analyses were undertaken within a linear or logistic regression framework with mixed-effects models utilised to accommodate multiple measures per individual or pair. Viral load was log transformed prior to analysis, and age was considered in models as a potential confounding factor. Correlation analyses utilised Spearman’s rho, and paired differences of IFN-γ responses were assessed using the Wilcoxon signed rank test. Subjects on ART were excluded from viral load analyses. Statistical analyses were performed using GraphPad Prism 8 and S-Plus (TIBCO Spotfire S+ 8.2 for Windows), with statistical significance threshold set at α = 0.05.

## Supporting information

S1 Table Number of adaptations examined by protein for each subject with those shared between mother and child in brackets. (XLSX)

S2 Table Peptide pool tested. (XLSX)

S3 Table HIV sequence content of mother/child pairs for tested peptide pools. (XLSX)

S4 Table *ERAP1* allotypes for mother/child pairs. (XLSX)

S5 Table Primer combinations for HLA genotyping by next-generation sequencing. (XLSX)

S6 Table PCR cycling conditions for next-generation sequencing and qPCR. (XLSX)

S7 Table Primer combinations for next-generation sequencing and qPCR with corresponding amplicon lengths. (XLSX)

S8 Table Sequencing summary information. (XLSX)

S1 Fig Phylogenetic analysis of HIV sequences of mother/child pairs confirms vertical transmission and greater genetic diversity in maternal quasispecies. Phylogenetic analysis strongly supports HIV transmission between mother/child pairs for Gag (A), Pol (B), and Nef (C), with no evidence of larger transmission networks within the cohort. The evolutionary history of the data was inferred using Maximum likelihood. The rate variation among sites was modelled with a gamma distribution (shape parameter = 0.72). Mother, M = closed diamond and child, C = open diamond. Intra-individual genetic diversity of Gag (D), Pol (E), and Nef (F) quasispecies was significantly higher in the mother than the child (paired t-test). Evolutionary analyses were conducted in MEGA7 [53]. Mean and standard error bars are shown. p<0.01 (**), p<0.001 (***). Note, for Pol and Nef only N = 23 mother/child pairs were sequenced, with N *=* 26 for Gag.
(TIF)

S2 Fig Most adaptations in the mother are fixed or near-fixed (≥90%) in the quasispecies population. The y-axis represents the number of adaptations in each mother dependent on the detection threshold shown on the x-axis. At 0% variant cut-off, an adaptation is considered present at any frequency within the quasispecies population. At a 5% cut-off, only those adaptations at or above 5% of the quasispecies population are considered and so on at 5% increments until fixation (100%). The difference in the number of adaptations scored per mother (on average) by increasing the threshold from 0% to 100% is approximately 5. Data are represented as mean ± SE.
(TIF)

S3 Fig Circulating adaptation scores are lower than the transmitted adaptation scores for maternal HLA alleles, but not paternal HLA alleles. Adaptation scores for a specific HLA allele were compared to their corresponding circulating adaptation score (the difference is plotted). Scores above zero indicate an adaptation score greater than the adaptation score for a ‘circulating’ HIV strain in the population. Comparison of adaptation versus circulating scores for maternal HLA-A p = 0.001, -B p = 0.0008, -C p = 0.2, and paternal HLA-A p = 0.5, -B p = 0.2, -C p = 0.9 (paired t-tests). Data are represented as mean ± SE. N = 33 for HLA-A, N = 37 for HLA-B, and N = 30 for HLA-C.
(TIF)

S4 Fig Comparison of the frequency of specific amino acids present in mother/child pairs. (A) All amino acids across the three proteins (N = 34,775), adapted amino acids in (B) Gag (N = 168), (C) Pol (N = 62) and (D) Nef (N = 403). Points are superimposed on each other and the color range reflects number of superimposed points with red (maximum, max) and blue (minimum, min).
(TIF)

S5 Fig Comparison of sites available for adaptation in the child based on maternally and paternally inherited HLA alleles. There was a significantly higher percentage of sites in the transmitted virus that did not have the adapted amino acid relevant to the paternal HLA allele than to the maternal HLA alleles in the transmitted virus (N *=* 26, p = 0.002; paired t-test). Data are represented as mean ± SE, p<0.01 (**).
(TIF)

S6 Fig *De novo* HIV adaptations confirmed in the child following transmission using ICS. Representative plot (of child 9) showing IFN-γ responses for unstimulated (A) and stimulation of cells (B) with a peptide pool that contained predicted T cell epitopes covering a *de novo* adaptation in the child.
(TIF)

S7 Fig Comparison of CD4^+^ T cell percentages between flow cytometry data and clinical data. (A) There was no significant difference between clinical CD4^+^ T cell % and flow cytometry CD4^+^ T cell % (N = 40, p<0.0001, r = 0.78; Spearman’s rho). (B) There was no significant difference between the two timepoints measured by flow cytometry (N = 24, p<0.0001, r = 0.84). 95% CI are shown.
(TIF)

S8 Fig Primer ID analysis of five subjects shows high concordance for amino acid frequencies. Frequency analysis shows a strong correlation between sequencing with (x-axis) and without (y-axis) primer ID (p<0.0001, r = 0.86; linear regression). The majority amino acid in the PID sequence is plotted with the corresponding percentage in the matching sequence. If only sites with variation from 100% are looked at the correlation remains significant (p<0.0001, r = 0.75; linear regression). 95% CI are shown.
(TIF)

S9 Fig Correlation of adaptation scores. Autologous adaptation scores correlate with adaptation scores calculated using an online tool developed by Carlson et al. [3] (N = 46, p = 0.0004, r = 0.5; Spearman’s rho). 95% CI are shown.
(TIF)
