# Supplementary figures and images for: Deep sequence analysis of HIV adaptation following vertical transmission reveals the impact of immune pressure on the evolution of HIV
Source: PLoS Pathog. 2019 Dec 10;15(12):e1008177. doi: 10.1371/journal.ppat.1008177 (PMC6924686; doi:10.1371/journal.ppat.1008177)

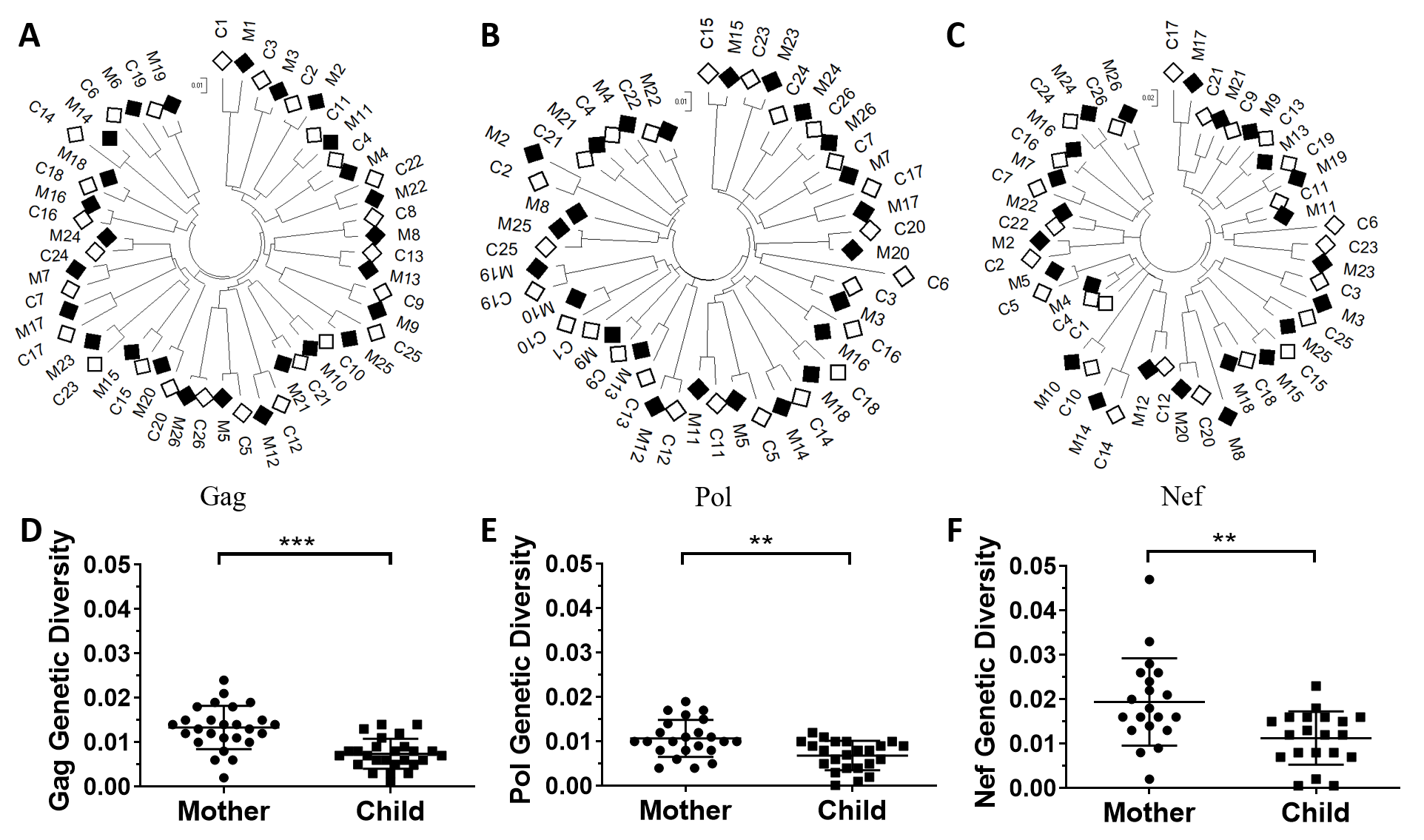

Supplement: S1 Fig — Phylogenetic analysis strongly supports HIV transmission between mother/child pairs for Gag (A), Pol (B), and Nef (C), with no evidence of larger transmission networks within the cohort. The evolutionary history of the data was inferred using Maximum likelihood. The rate variation among sites was modelled with a gamma distribution (shape parameter = 0.72). Mother, M = closed diamond and child, C = open diamond. Intra-individual genetic diversity of Gag (D), Pol (E), and Nef (F) quasispecies was significantly higher in the mother than the child (paired t-test). Evolutionary analyses were conducted in MEGA7 [53]. Mean and standard error bars are shown. p<0.01 (**), p<0.001 (***). Note, for Pol and Nef only N = 23 mother/child pairs were sequenced, with N = 26 for Gag. (TIF) [file ppat.1008177.s009.tif]

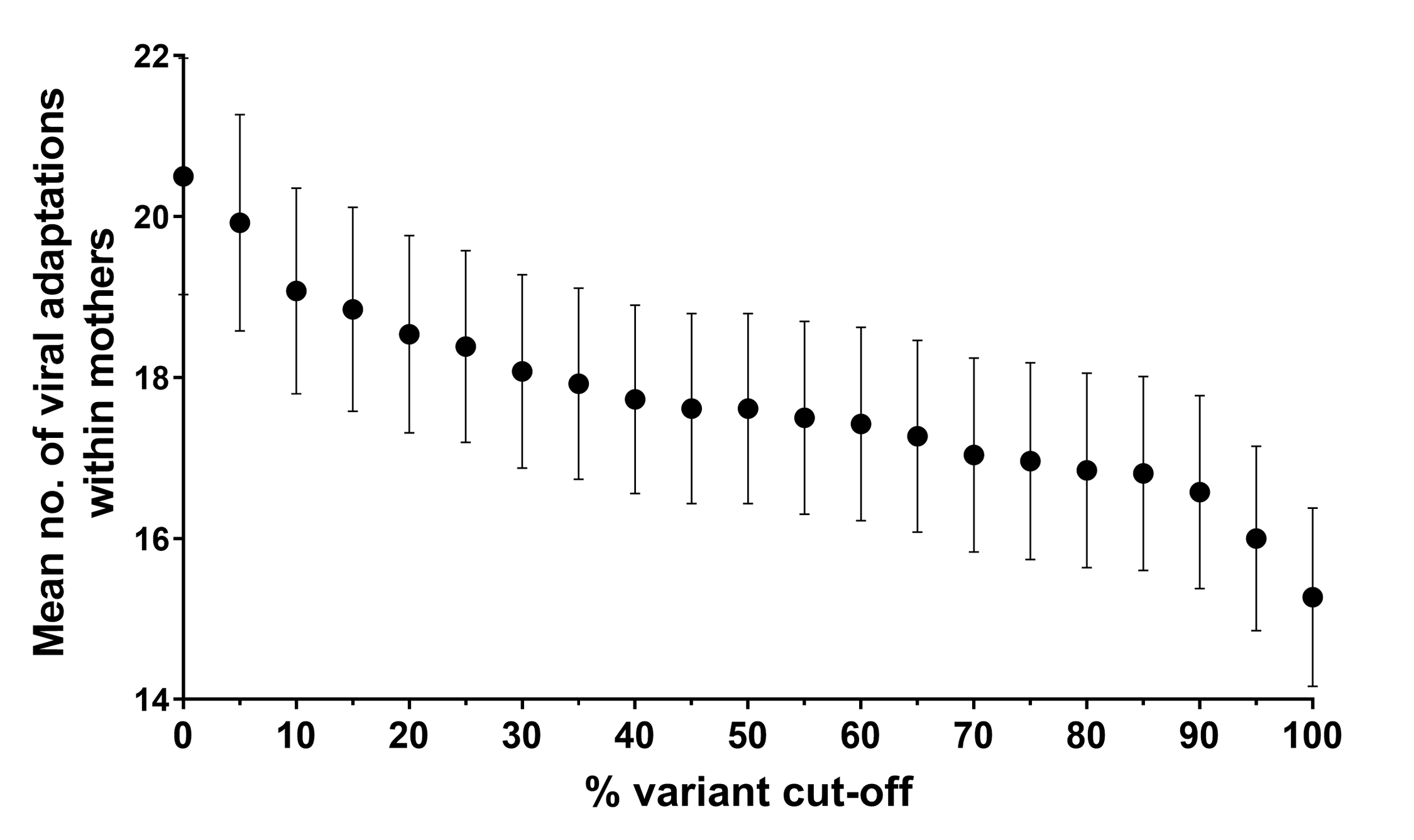

Supplement: S2 Fig — The y-axis represents the number of adaptations in each mother dependent on the detection threshold shown on the x-axis. At 0% variant cut-off, an adaptation is considered present at any frequency within the quasispecies population. At a 5% cut-off, only those adaptations at or above 5% of the quasispecies population are considered and so on at 5% increments until fixation (100%). The difference in the number of adaptations scored per mother (on average) by increasing the threshold from 0% to 100% is approximately 5. Data are represented as mean ± SE. (TIF) [file ppat.1008177.s010.tif]

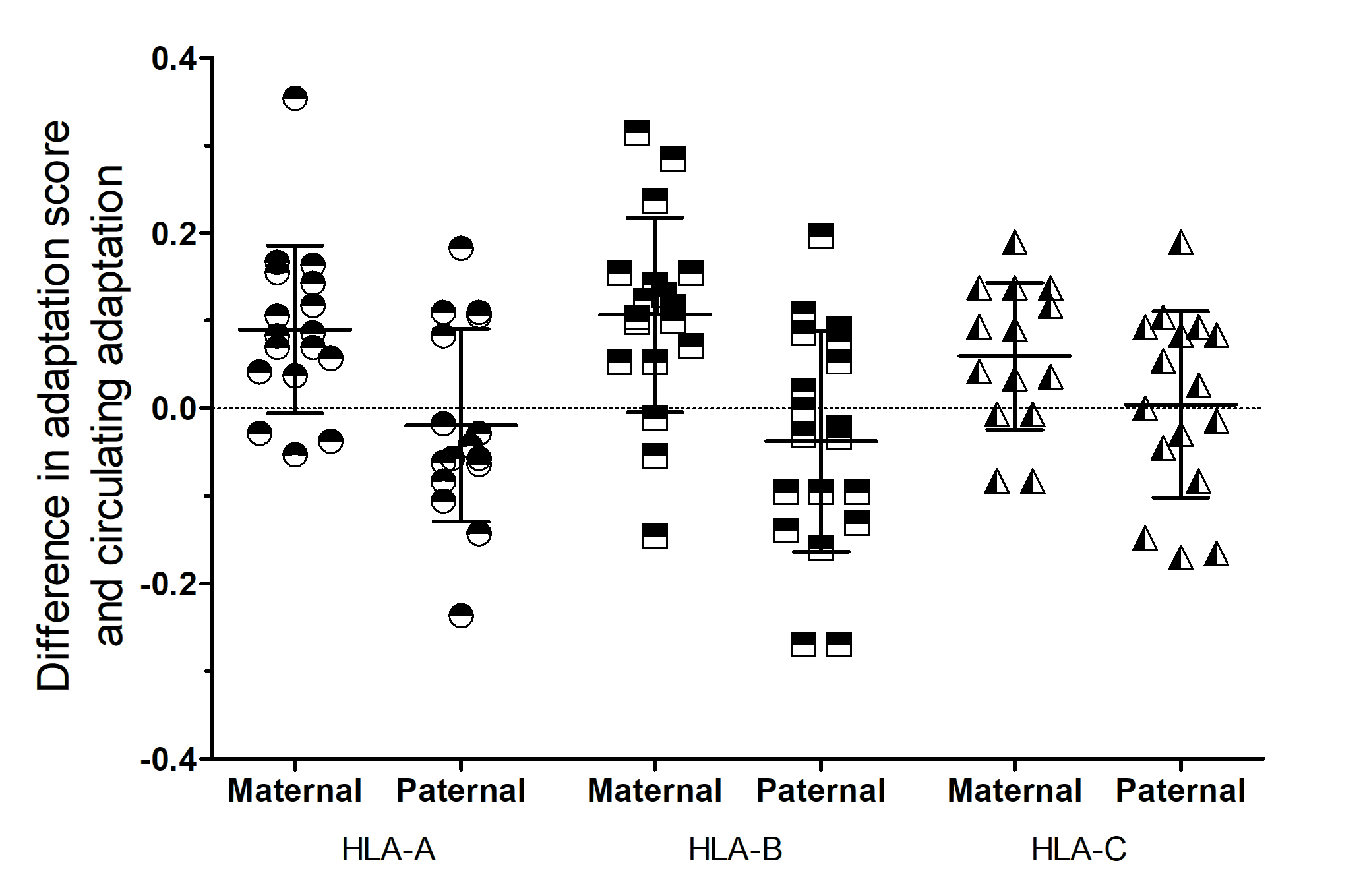

Supplement: S3 Fig — Adaptation scores for a specific HLA allele were compared to their corresponding circulating adaptation score (the difference is plotted). Scores above zero indicate an adaptation score greater than the adaptation score for a ‘circulating’ HIV strain in the population. Comparison of adaptation versus circulating scores for maternal HLA-A p = 0.001, -B p = 0.0008, -C p = 0.2, and paternal HLA-A p = 0.5, -B p = 0.2, -C p = 0.9 (paired t-tests). Data are represented as mean ± SE. N = 33 for HLA-A, N = 37 for HLA-B, and N = 30 for HLA-C. (TIF) [file ppat.1008177.s011.tif]

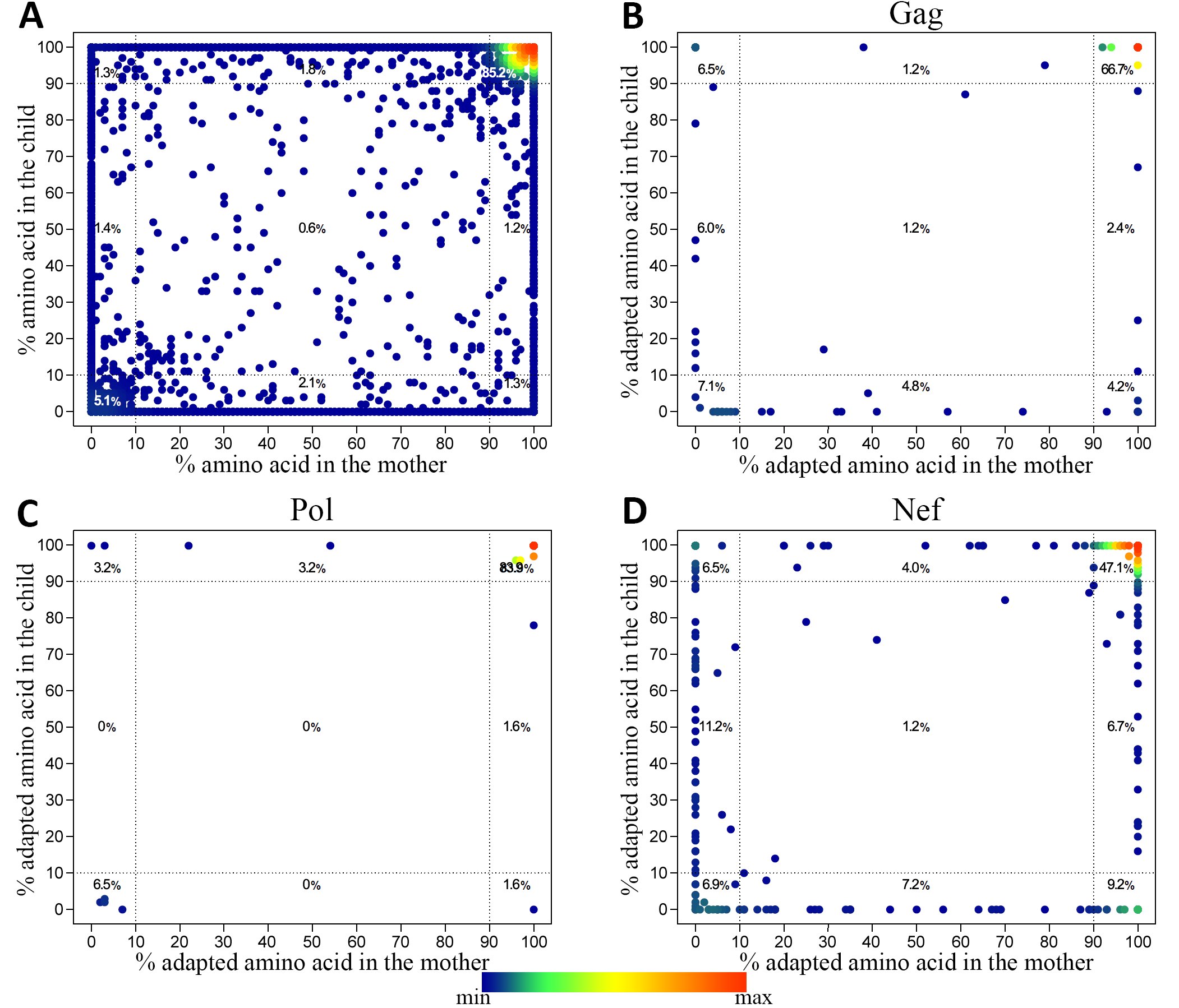

Supplement: S4 Fig — (A) All amino acids across the three proteins (N = 34,775), adapted amino acids in (B) Gag (N = 168), (C) Pol (N = 62) and (D) Nef (N = 403). Points are superimposed on each other and the color range reflects number of superimposed points with red (maximum, max) and blue (minimum, min). (TIF) [file ppat.1008177.s012.tif]

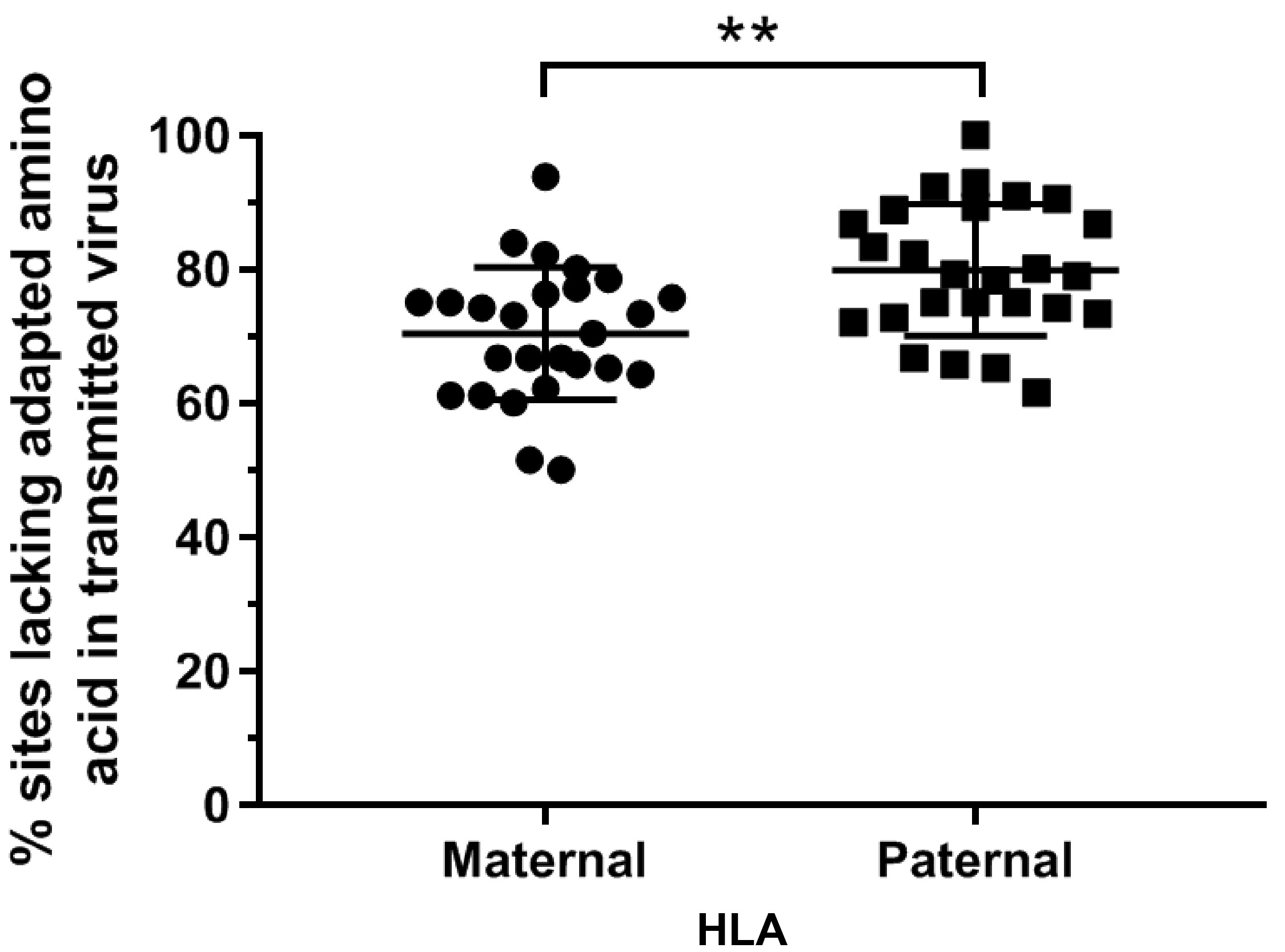

Supplement: S5 Fig — There was a significantly higher percentage of sites in the transmitted virus that did not have the adapted amino acid relevant to the paternal HLA allele than to the maternal HLA alleles in the transmitted virus (N = 26, p = 0.002; paired t-test). Data are represented as mean ± SE, p<0.01 (**). (TIF) [file ppat.1008177.s013.tif]

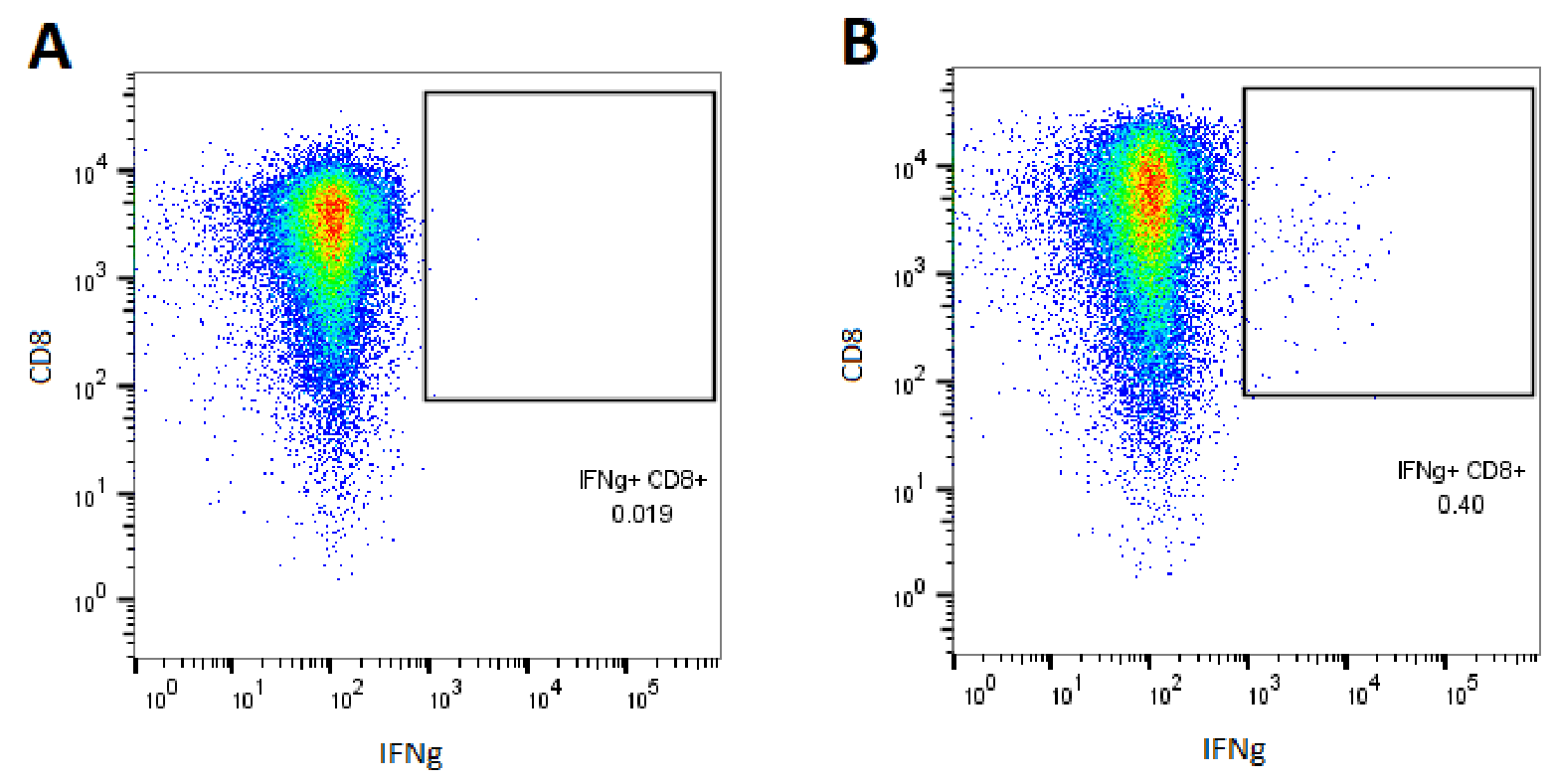

Supplement: S6 Fig — Representative plot (of child 9) showing IFN-γ responses for unstimulated (A) and stimulation of cells (B) with a peptide pool that contained predicted T cell epitopes covering a de novo adaptation in the child. (TIF) [file ppat.1008177.s014.tif]

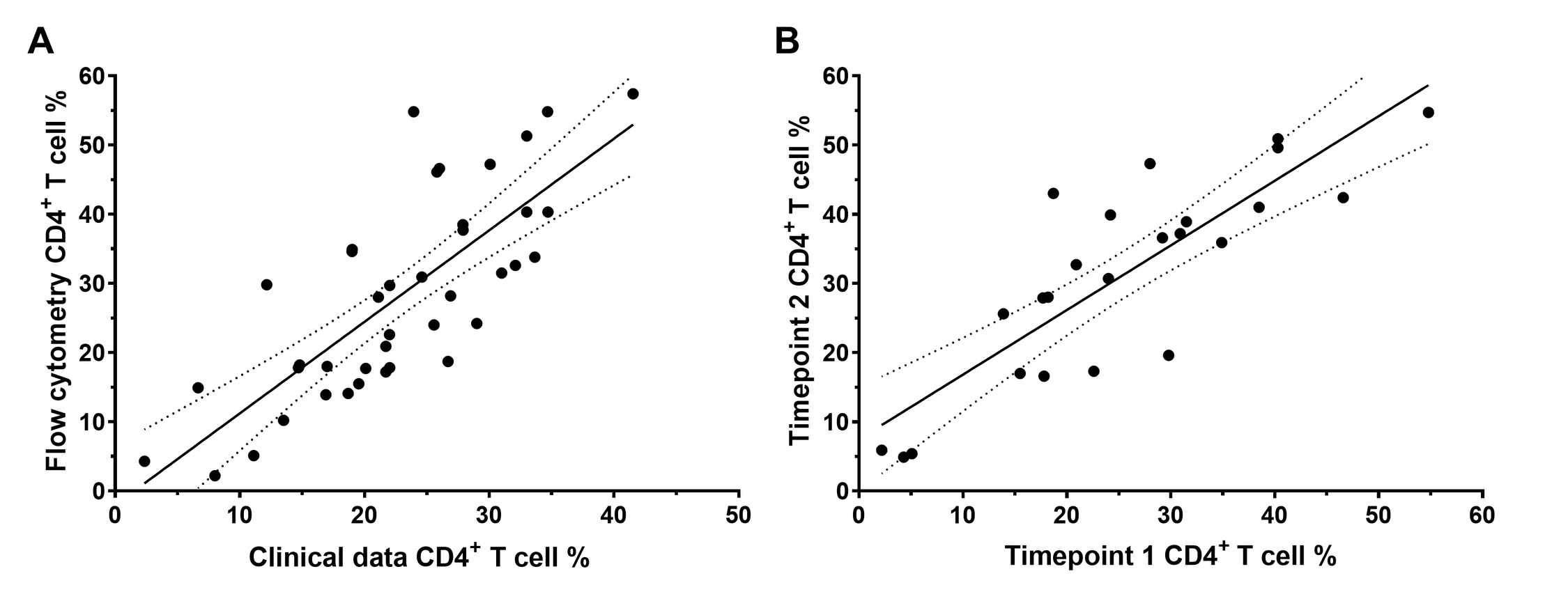

Supplement: S7 Fig — (A) There was no significant difference between clinical CD4+ T cell % and flow cytometry CD4+ T cell % (N = 40, p<0.0001, r = 0.78; Spearman’s rho). (B) There was no significant difference between the two timepoints measured by flow cytometry (N = 24, p<0.0001, r = 0.84). 95% CI are shown. (TIF) [file ppat.1008177.s015.tif]

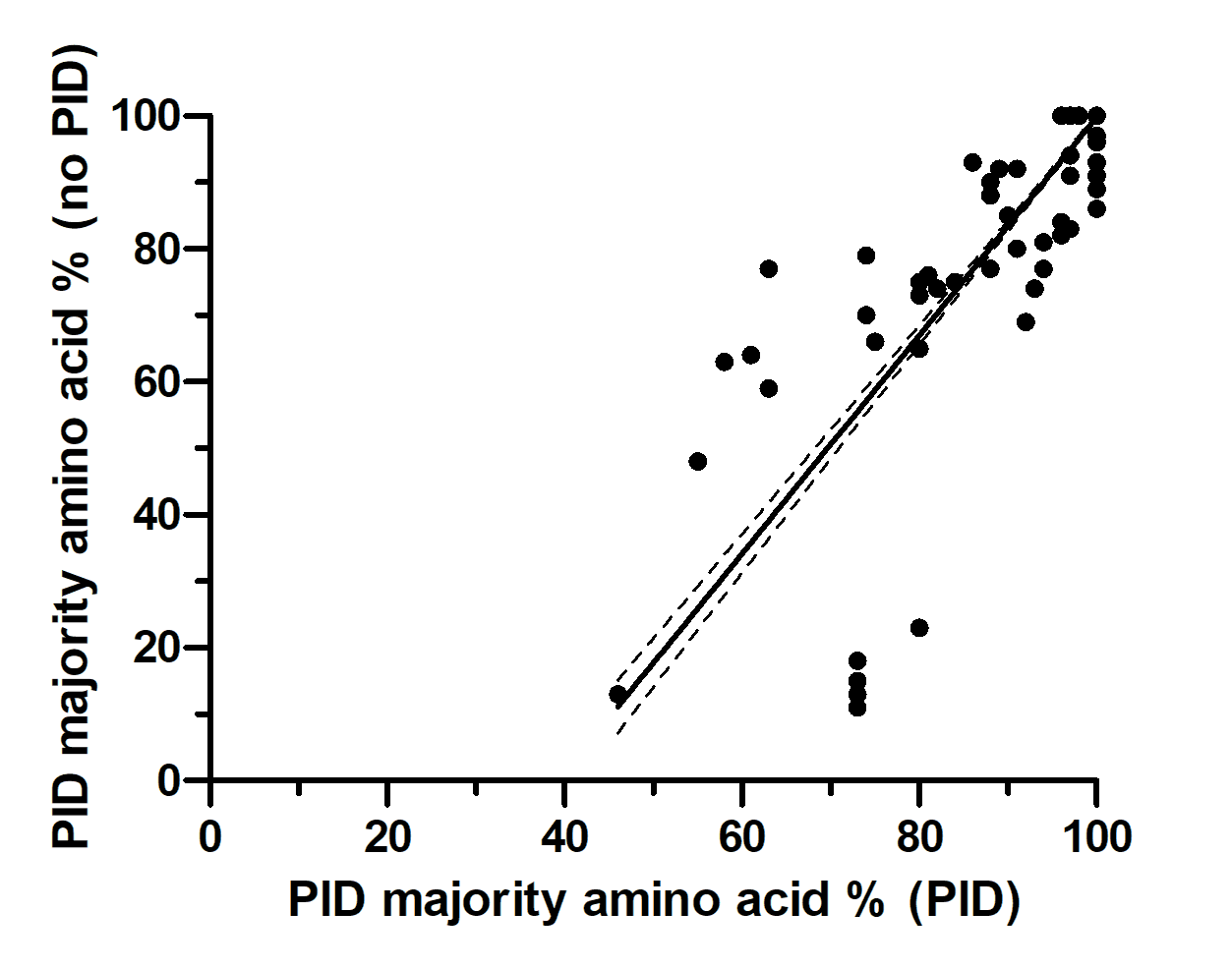

Supplement: S8 Fig — Frequency analysis shows a strong correlation between sequencing with (x-axis) and without (y-axis) primer ID (p<0.0001, r = 0.86; linear regression). The majority amino acid in the PID sequence is plotted with the corresponding percentage in the matching sequence. If only sites with variation from 100% are looked at the correlation remains significant (p<0.0001, r = 0.75; linear regression). 95% CI are shown. (TIF) [file ppat.1008177.s016.tif]

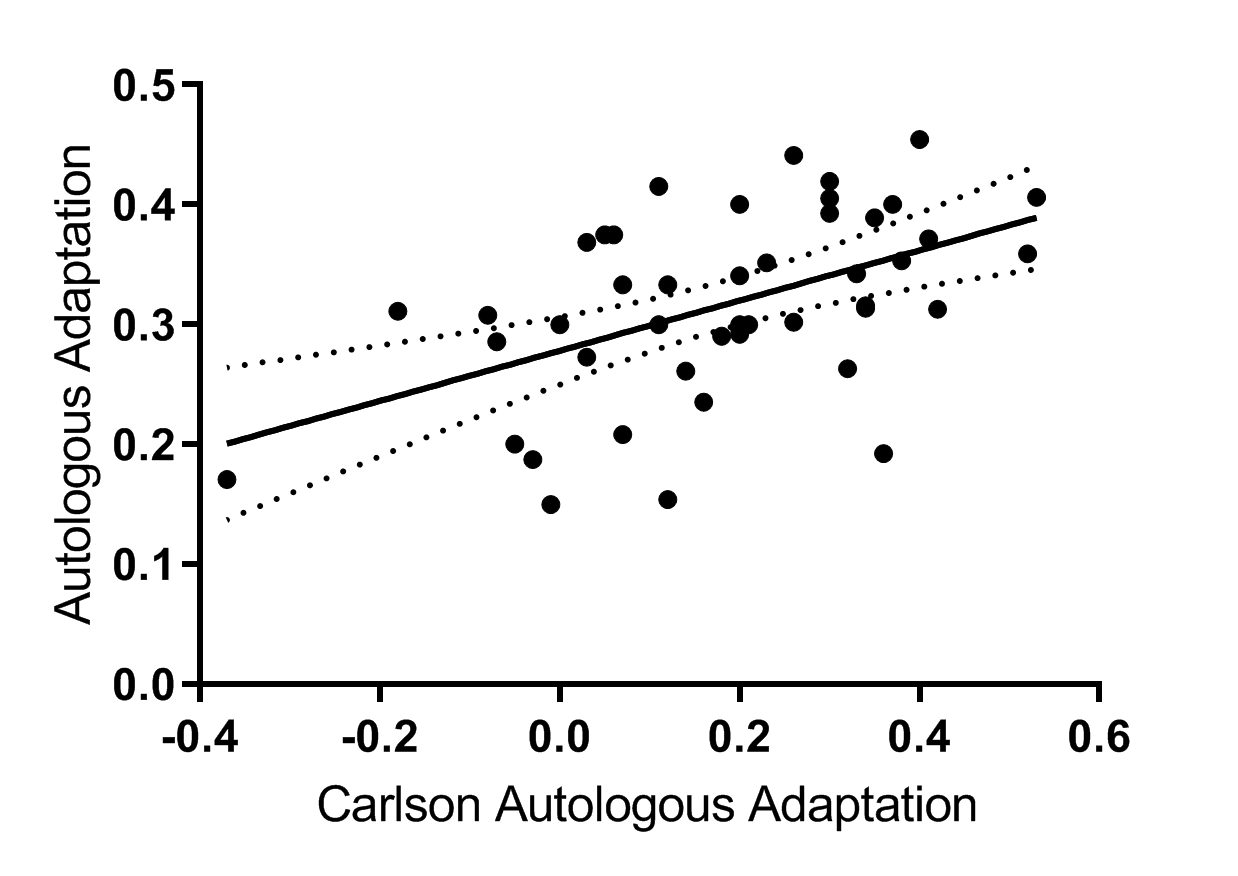

Supplement: S9 Fig — Autologous adaptation scores correlate with adaptation scores calculated using an online tool developed by Carlson et al. [3] (N = 46, p = 0.0004, r = 0.5; Spearman’s rho). 95% CI are shown. (TIF) [file ppat.1008177.s017.tif]
